# Orbital‐Hybridizable Nanoseed Interphase Enables One‐Minute Rechargeable, Energy‐Dense Anode‐Free Aqueous Zinc Batteries

**DOI:** 10.1002/adma.73553

**Published:** 2026-05-29

**Authors:** Won‐Yeong Kim, Ahyeon Son, Ohchan Kwon, Suseong Hyun, Sung Jun Hong, Hong‐I Kim, Ju Yeon Kim, Hyunseo Kang, Seung‐Hyeok Kim, Xu Liu, Kyeong‐Seok Oh, Jee Ho Ha, Seok Ju Kang, Stefano Passerini, Byungchan Han, Dae Woo Kim, Sang‐Young Lee

**Affiliations:** ^1^ Department of Chemical and Biomolecular Engineering Yonsei University Seoul Republic of Korea; ^2^ Department of Chemistry University of California Berkeley Berkeley California USA; ^3^ Department of Battery and Chemical Engineering Hanyang University ERICA Gyeonggi Republic of Korea; ^4^ Helmholtz Institute Ulm (HIU) Ulm Germany; ^5^ Karlsruhe Institute of Technology (KIT) Karlsruhe Germany; ^6^ Department of Energy Engineering School of Energy and Chemical Engineering Ulsan National Institute of Science and Technology (UNIST) Ulsan Republic of Korea

**Keywords:** anode‐free Zn batteries, aqueous electrolytes, graphene oxide nanoribbons, one‐minute recharging, orbital‐hybridizable nanoseed interphase

## Abstract

Aqueous zinc (Zn) batteries are emerging as promising candidates for energy storage systems (ESS) and wearable electronics, but their practical application is hindered by low energy density and electrochemical instability at the Zn anode–electrolyte interface. Here, we report an orbital‐hybridizable nanoseed (OHNS) interphase composed of graphene oxide nanoribbons (GONRs) uniformly deposited on Cu current collectors via a scalable slot‐die coating process, enabling one‐minute‐rechargeable, energy‐dense anode‐free aqueous Zn batteries. The carbon edges (C‐edges) prevalent in the GONR facilitate orbital hybridization with Zn. This chemical interplay between the heteroatoms (C‐edge and Zn) enhances Zn nucleation kinetics and retards surface diffusion of adsorbed Zn, thereby promoting corrosion‐resistant, (002)‐oriented growth of Zn. Consequently, reversible Zn plating/stripping with high Coulombic efficiency (∼99.5%) was achieved even at a high current density of 120 mA cm^−2^. Moreover, anode‐free full cells with the OHNS interphase delivered a maximum energy/power density of 140.6 Wh kg^−1^/4138.1 W kg^−1^. Notably, anode‐free pouch cells exhibited stable capacity retention of 82.2% after 800 cycles at a fast charge/discharge current density of 106C (equivalent to a time of 34 s).

## Graphical abstract

An orbital‐hybridizable nanoseed (OHNS) interphase induces strong orbital hybridization between Zn and carbon edges, accelerating Zn nucleation while suppressing 2D surface diffusion. This interfacial electronic regulation drives uniform, dense, and (002)‐oriented Zn growth, enabling stable and fast charging behavior in anode‐free aqueous Zn batteries.

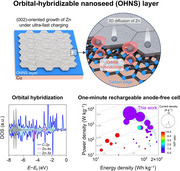

## 1 Introduction

The ever‐increasing demand for safe, sustainable, and fast‐charging batteries drives the ongoing pursuit of advanced energy storage systems beyond commercial Li‐ion batteries [1, 2, 3, 4]. Aqueous Zn batteries are emerging as promising candidates for use in stationary energy storage systems (ESS) and wearable electronics [5, 6, 7], owing to the natural abundance of Zn, high specific capacity (820 mAh g_Zn_^−1^), low redox potential, intrinsic safety, and low‐cost (dry room‐free) cell manufacturing [8, 9]. Moreover, utilizing aqueous electrolytes contributes to higher ionic conductivity [10, 11] and lower energy barriers for redox reactions [12, 13] compared to their organic counterparts.

Despite their potential, the realization of fast‐chargeable aqueous Zn batteries is limited by the interfacial side reactions of Zn anodes in contact with aqueous electrolytes [14, 15, 16, 17, 18]. This challenge is further exacerbated in anode‐free cell configurations [19], which attempt to maximize cell energy densities by employing only a current collector without Zn metal. During charging of the anode‐free cells, nucleation of Zn initially occurs on a Cu current collector, followed by growth of Zn on the preformed Zn nuclei. However, inhomogeneous Zn nucleation forms on the Cu current collector because of the slow kinetics of Zn nucleation [20]. Subsequently, two‐dimensional (2D) surface diffusion of adsorbed Zn atoms toward energetically favorable nucleation sites takes place to minimize the surface energy [21]. Hence, dendritic growth of Zn along a (101) plane is promoted [22], resulting in a random and uneven porous structure. This structural inhomogeneity accelerates side reactions at Zn anode‐electrolyte interfaces, such as chemical corrosion of Zn and hydrogen gas evolution [23, 24, 25].

Substantial efforts have been implemented to address the above‐described issues, including the formation of stable solid electrolyte interphase by addition of organic compounds [19, 26, 27, 28, 29], the application of protective layers [30, 31, 32, 33], and the design of host structures [34, 35, 36]. However, most of these approaches have limitations in improving the fast‐charging capability of the resulting cells, mainly because of the elevated polarization. In particular, many substrate modification approaches rely on physical coating techniques such as drop casting [37, 38, 39], spray coating [40, 41, 42], and doctor blading [43, 44, 45], which often yield thick layers that hinder ion transport and thereby limit high‐rate performance [46] (Table S1). Alternative methods, including chemical vapor deposition [47] and solvothermal processes [48], require high costs or elevated temperatures, reducing their feasibility for practical applications [46]. Moreover, the flammable nature of the incorporated components within the cell impairs both the economic and environmental benefits of aqueous Zn cells [49], and the challenges associated with large‐area fabrication of these modified interfaces pose barriers to their practical applications [50].

**FIGURE 1:**
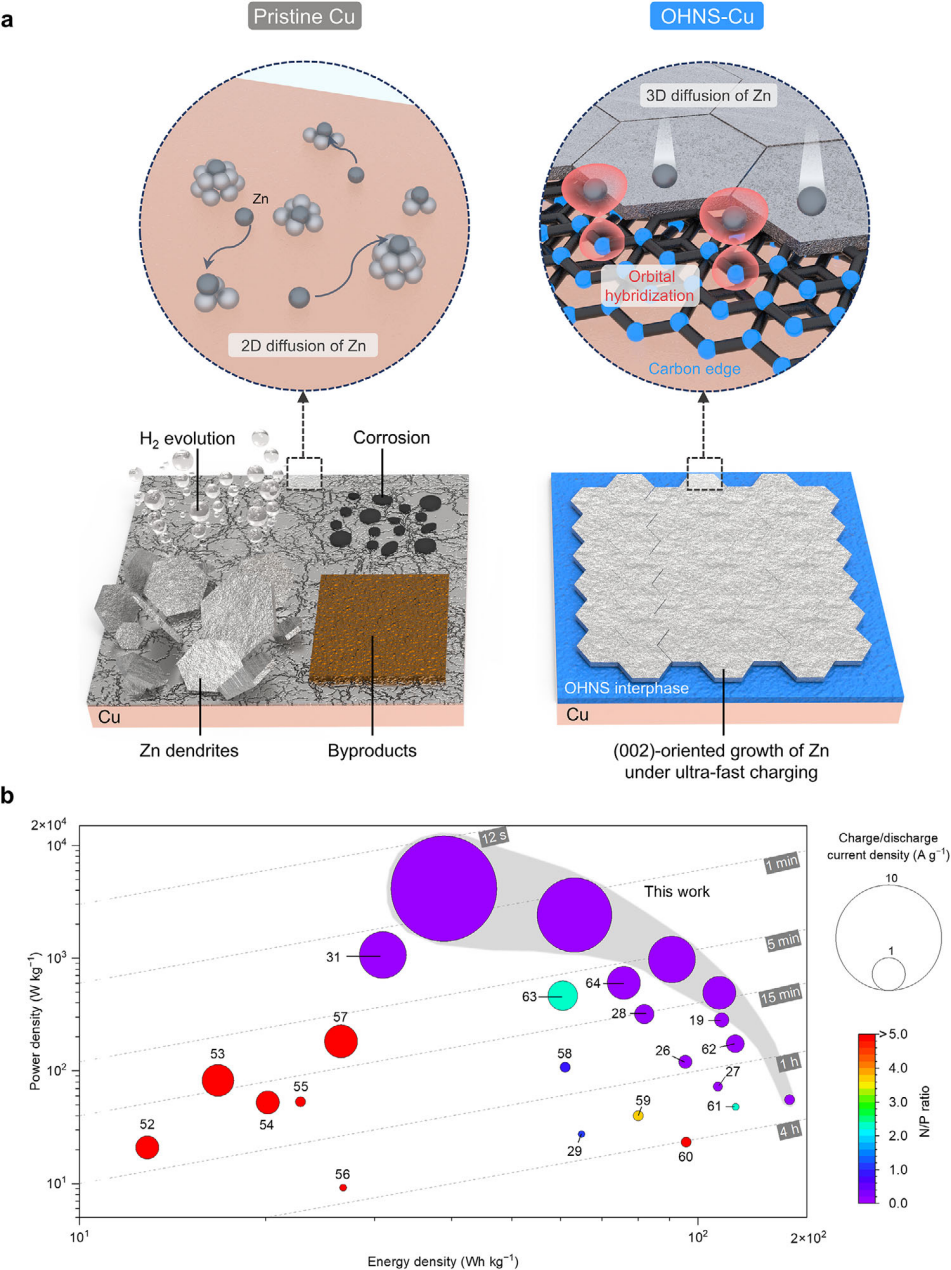
GONR‐based OHNS interphase for ultrafast‐rechargeable, energy‐dense anode‐free aqueous Zn batteries. (a) Schematic of interfacial side reaction during Zn electrodeposition (Cu foil (left) vs. OHNS‐Cu foil (right)). (b) Comparison of cell performance between this study and previously reported aqueous Zn batteries, focusing on energy density (*x*‐axis), power density (*y*‐axis), charge/discharge current density (represented by area), and N/P ratio (indicated by heatmap). The gray dashed lines denote the charging/discharging time. Energy and power densities are calculated based on the total mass of electrodes.

To achieve the goal of fast‐rechargeable anode‐free aqueous Zn batteries with high energy density, herein, we present an orbital‐hybridizable nanoseed (OHNS) interphase strategy based on graphene oxide nanoribbons (GONRs). The GONRs are synthesized via longitudinal unzipping of multi‐walled carbon nanotubes (MWNTs), yielding a one‐dimensional morphology with a higher density of carbon edges (C‐edges) compared to the basal plane. This unique structural feature fundamentally alters the physicochemical and electrochemical properties of GONRs, distinguishing them from conventional two‐dimensional graphene oxide (GO) flakes [51]. Departing from conventional coating techniques, we adopt a slot‐die coating process, commonly used in roll‐to‐roll manufacturing, as a scalable and industrially viable method for depositing GONRs on Cu foil. The prevalence of C‐edges in GONR facilitates orbital hybridization with 3*d* orbitals of Zn. This chemical interplay between the heteroatoms (C‐edge and Zn) facilitated the Zn nucleation kinetics and simultaneously inhibited the 2D surface diffusion of adsorbed Zn (Figure 1a), promoting the growth of homogeneous, dense, and (002)‐textured Zn. Hence, reversible Zn plating/stripping with high Coulombic efficiency (∼99.5%) was achieved at a high current density of 120 mA cm^−2^ while suppressing water‐triggered interfacial side reactions (such as H_2_ evolution and Zn corrosion) in aqueous electrolytes.

This OHNS‐deposited Cu (OHNS‐Cu) was paired as an anode with a pre‐zincificated cathode (Zn*_x_* CaV_6_O_16_, Zn*_x_* CVO) to produce an energy‐dense anode‐free full cell (OHNS‐Cu||Zn*_x_* CVO). The resulting anode‐free full cells achieved a maximum energy/power density of 140.6 Wh kg^−1^/4138.1 W kg^−1^ based on the total mass of electrodes. Moreover, anode‐free pouch cells exhibited stable capacity retention of 82.2% after 800 cycles at a fast charge/discharge current density of 106C (equivalent to a time of 34 s). This cell performance enabled by the OHNS interphase far surpassed those of previously reported aqueous Zn batteries [19, 26, 27, 28, 29, 31, 52, 53, 54, 55, 56, 57, 58, 59, 60, 61, 62, 63, 64] (Figure 1b, Tables S2 and S3), in which the energy and power densities were estimated based on the total mass of both electrodes (see Table S4 for calculation details). This comparison highlights the effectiveness of the orbital hybridization in stabilizing the Zn plating/stripping behavior, contributing to the development of one‐minute rechargeable, energy‐dense anode‐free aqueous Zn batteries.

## 2 Results and Discussion

### 2.1 Scalable Fabrication and Characterization of GONR‐Deposited Cu Foil

**FIGURE 2:**
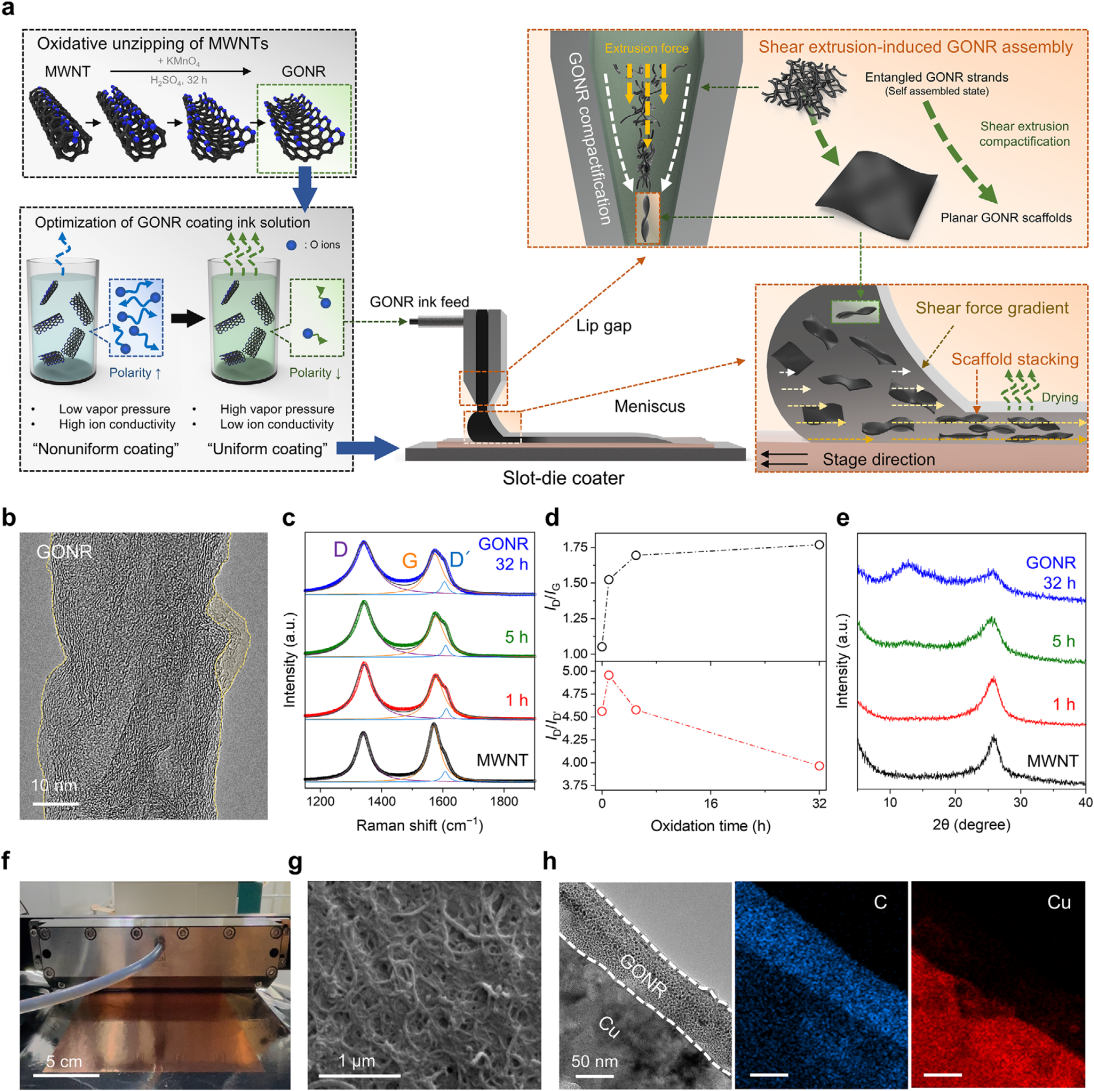
Scalable fabrication and characterization of GONR‐deposited Cu foil. (a) Schematic of scalable fabrication process of GONR‐deposited Cu foil utilizing a slot‐die coater. (b) TEM image of GONR strand with layered unzipped graphene planes, in which yellow contours mark unzipped edges. (c) Raman spectra of MWNTs as a function of oxidation time and (d) corresponding D/G (top), and D/D’ (bottom) ratios derived from fitted Raman spectra. (e) XRD patterns of MWNTs as a function of oxidation time. (f) Photograph of a scalable slot‐die coater capable of producing GONR‐deposited Cu foils with dimensions up to 15 × 15 cm^2^. (g) Surface SEM image of GONR‐deposited Cu foil. (h) Cross‐sectional TEM image and EDS mappings of GONR‐deposited Cu foil.

The scalable fabrication process of the GONR‐deposited Cu foil is illustrated in Figure 2a, details of which are described below. The GONRs were synthesized by longitudinal unzipping of MWNTs utilizing a strong oxidant (potassium permanganate, KMnO_4_) for 32 h [65, 66, 67]. The transmission electron microscopy (TEM) image displayed the morphology of the resulting unzipped GONR strand with layered edge structures (Figure 2b). Compared to the MWNTs, the lattice structures of the basal plane were obscured owing to the presence of oxygen groups and defects induced by oxidation. The expansion of the strand width of the GONR was attributed to the exposure of the unzipped graphene planes [66, 68] (Figure S1).

Different samples were prepared by varying the oxidation time (0, 1, 5, and 32 h) to investigate the relationship between the extent of unzipping and the density of C‐edges. The Raman spectra were utilized to characterize defects and edge structures in both the MWNTs and GONRs (Figure 2c). Each Raman spectrum was deconvoluted into three distinct bands at 1340, 1575, and 1610 cm^−1^, corresponding to D, G, and D’, respectively. The D band, indicative of the *A*_1g_ breathing mode of the carbon ring, correlates with the structural defects. Meanwhile, the G band reflects the *E*_2g_ mode of in‐plane vibration of the carbon lattice and is proportional to the *sp* ^2^ hybridized carbon bonds [69, 70, 71]. As the oxidation level increased, a rise in the intensity of the D band (*I*_D_) was observed, along with a broadening when compared to the G band (*I*_G_). This resulted in an increase of *I*_D_/*I*_G_ ratio from 1.05 for the MWNTs to 1.77 for the GONRs (Figure 2d), indicating a higher density of defects and disorder in the GONRs. In addition, the D’ band, which is associated with the *E*_2g_ stretching mode of the surface graphene layers, is influenced by disruption in the symmetry due to the functional groups or edge carbons, linking it closely to the nature of the defects, particularly as the *I*_D_/*I*_D’_ ratio [72, 73, 74, 75, 76]. An *I*_D_/*I*_D’_ value of 3.96 for the GONRs indicates predominant boundary defects, which are attributed to the high density of boundary and edge carbons in the unzipped morphology of the GONR.

The X‐ray photoelectron spectroscopy (XPS) was employed to analyze the change in chemical structure after the oxidation [77] (Figure S2). A significant increase in the contribution of the C═O bond was observed at the GONR compared to the pristine MWNT. This bond is known to typically localize at the graphene edges as carbonyl and carboxyl groups, indicating an enriched density of C‐edges in the GONR [68, 78]. Furthermore, the decrease in electrical conductivity reveals an increased degree of functionalization, decreasing the domains of *sp* ^2^ hybridized carbon (Figure S3). The X‐ray diffraction (XRD) patterns were obtained to explore the microdomain structures at different degrees of oxidation (Figure 2e). The high‐angle peak at 25.6° is indicative of diffraction from the intramolecular graphene layers within the multiwall structure of the MWNTs [79]. Upon oxidation, incorporating functional groups disrupts these stacked layers, manifested by the broadening of the peak. Moreover, a new lower‐angle peak at 12.6° was observed, which is attributed to the intermolecular stacking of the GONR layers [80]. This comprehensive analysis of the TEM, Raman, XPS, and XRD data confirms the successful synthesis of GONRs, characterized by an increased density of edge carbons compared to the MWNTs.

Typical coating techniques such as vacuum filtration, spin coating, and spray coating are commonly used to deposit carbon layers on substrates. In contrast, we exploited a slot‐die coater, utilized for continuous production of polymer films [81, 82], as a scalable approach to deposit the GONR layers on a Cu foil. Unlike conventional techniques, slot‐die coating allows for low concentration, less‐viscous solutions while maintaining coating uniformity. The coating process relies on a liquid meniscus formed at the interface between the substrate and the extrusion head, eliminating direct contact and mechanical disturbances. Although shear‐based coating methods benefit from the shear‐thinning behavior of viscoelastic solutions, conventional techniques such as rod and doctor blade coating fail to generate sufficient shear stress, necessitating the use of highly concentrated, gel‐like graphene oxide dispersions. In contrast, the micro‐gap of the slot die head provides significantly higher shear stress, facilitating the extrusion of uniform, sheet‐like scaffolds that conform closely to the Cu surface without defects (right panels of Figure 2a and Figure S4). This approach also enables precise control of the coating layer thickness by simply adjusting the concentration of the GONR dispersion (here, 2.5 mg mL^−1^ in acetone). A photograph of the slot‐die coater, capable of producing GONR‐deposited Cu foils with dimensions up to 15 × 15 cm^2^, is shown in Figure 2f. The scalability of this fabrication process is demonstrated in Video S1. Notably, under identical slot‐die coating conditions, precursor MWNT inks failed to produce a uniform and continuous coating on Cu, highlighting the improved processability of unzipping‐derived GONRs (Figure S5).

The Cu foil tends to oxidize when exposed to aqueous GONR ink (Figure S6). The presence of acidic residues in the aqueous ink can induce oxidation of the Cu foil surface even after extensive cleaning. This oxidation process was further exacerbated by the spontaneous reduction of GONRs, which could transfer oxygen species to the Cu because of the lower reduction potential [83]. To mitigate the Cu oxidation, it is proposed to replace water with low‐polarity solvents, which exhibit reduced ionic conductivity and thus alleviate the environment conducive to ion transfer. In addition, solvents with high volatility are recommended to minimize ion transfer while maintaining high production speed (bottom left panel of Figure 2a). Based on these criteria, acetone was selected as a suitable solvent for the GONR ink because of its low ionic conductivity, adequate dispersibility, high volatility, and economic feasibility among the solvents examined (Figure S7).

The scanning electron microscopy (SEM) and energy‐dispersive X‐ray spectroscopy (EDS) analyses exhibited the uniform deposition of an ultrathin GONR layer on the Cu foil (Figure 2g and Figure S8). The thickness of the GONR layer was confirmed to be approximately 80 nm through cross‐sectional TEM and EDS analyses (Figure 2h). This optimal thickness of the GONR layer was determined based on the coating uniformity at different solution concentrations (Figure S9) and their corresponding electrochemical performance (Figure S10). The GONR layer exhibited the lowest surface roughness among the oxidized MWNTs, attributed to the dense packing of the unzipped graphene sheets (Figure S11). The nitrogen (N_2_) adsorption–desorption isotherms (at 77 K) of the GONR indicated a microporous structure. In contrast, larger pores were identified in the MWNTs and less oxidized carbon materials (Figure S12). These results demonstrate the viability of the GONR in forming uniform and dense coating layers on the Cu foil.

### 2.2 Effect of Orbital Hybridization on Nucleation and Diffusion of Zn

**FIGURE 3:**
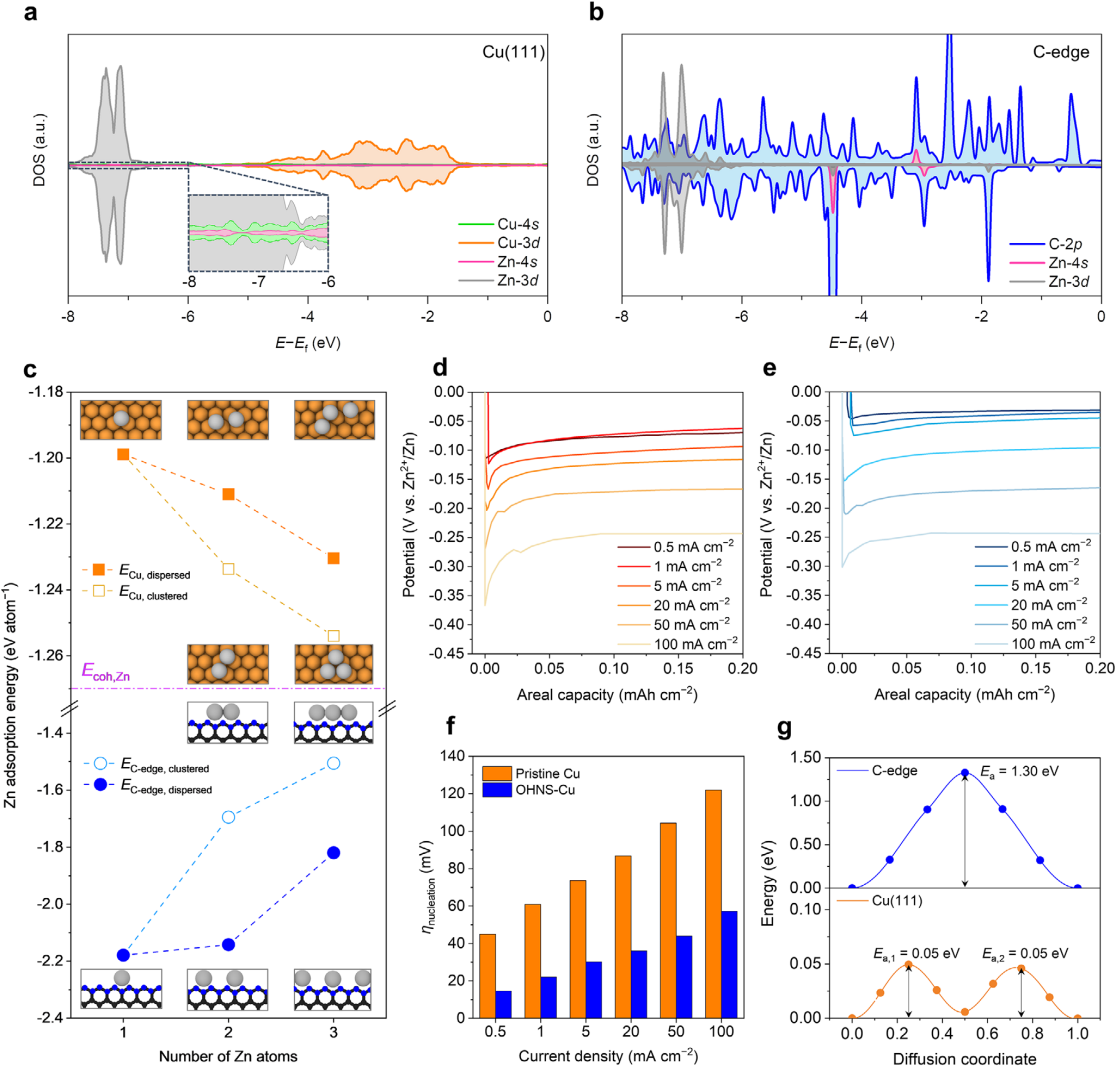
Effect of the orbital hybridization on nucleation and diffusion of Zn. pDOS spectra of Zn atom adsorbed on (a) Cu(111) and (b) C‐edges. The magnified pDOS of Cu(111) is shown in the inset of (a). (c) Thermodynamic adsorption energies of Zn atom(s) on Cu(111) and C‐edge sites, calculated to elucidate the atomic topology of adsorbed Zn atoms on the surface and edges. Voltage profiles of Zn deposition in Zn||Cu cells on (d) pristine Cu and (e) OHNS‐Cu at various current densities (0.5, 1, 5, 20, 50, and 100 mA cm^−2^). (f) Comparison of nucleation overpotential between pristine Cu and OHNS‐Cu. (g) Energy profiles of Zn diffusion on Cu(111) surface and GONR C‐edge, determined from NEB calculation.

To investigate the Zn deposition behavior on the GONR, the adsorption energies of Zn atoms on different sites (Cu(111), C‐basal, C‐edge, and oxygen‐containing functional groups in the GONR) are calculated utilizing density functional theory (DFT) (Figures S13 and S14). The results showed Zn atoms can thermodynamically adsorb on the functional sites on GONRs as well as the Cu(111) surface. Among them, the C‐edge exhibited the most favorable adsorption with the highest binding energy, surpassing those of the C‐basal, oxygen‐containing groups and the Cu surface. These results were corroborated by Zn^2+^ adsorption experiments on MWNTs with controlled C‐edge density, quantified by inductively coupled plasma optical emission spectroscopy (ICP‐OES) (Figure S15). To elucidate the mechanism underlying the adsorption energy difference, we theoretically analyzed the electronic interactions between Zn and each substrate, using partial density of states (pDOS) calculations. As the Zn atom has the electronic configuration of 1*s* ^2^ 2*s* ^2^ 2*p* ^6^ 3*s* ^2^ 3*p* ^6^ 3*d* ^10^ 4*s* ^2^, the change in the electronic state of the outermost 4*s* orbital and underlying filled 3*d* orbitals plays an important role in its physicochemical properties [78, 79]. For Zn adsorption on the Cu(111) plane, orbital hybridization between Cu‐3*d* and Zn‐3*d* orbitals is marginal, with only a weak peak observed for their 4*s* orbitals near the 3*d* orbital peaks (Figure 3a), suggesting limited orbital hybridization. Similarly, the pDOS spectra of C in the C‐basal site exhibited sharp peaks for the C‐2*p* orbitals that were non‐overlapped from the Zn orbitals, indicating the negligible orbital hybridization (Figure S16). In contrast, for the C‐edge, the 3*d* orbitals of Zn were broadened and overlapped with the 2*p* orbitals of the adjacent C atom, exhibiting the orbital hybridization between the Zn atom and the C‐edge in the GONR (Figure 3b). The degree of hybridization was intensified as the number of Zn atoms adsorbed on the C‐edge increased from two to four, suggesting that strong orbital hybridization was maintained (Figure S17).

These differences in the orbital hybridization behavior can be attributed to the electronic configurations of Zn, Cu, and the C‐edge of GONRs. _30_Zn possesses a fully occupied 4*s* ^2^ orbital, whereas _29_Cu (1*s* ^2^ 2*s* ^2^ 2*p* ^6^ 3*s* ^2^ 3*p* ^6^ 4*s* ^1^ 3*d* ^10^) has only one electron in its 4*s* orbital. This electronic configuration strengthens the intra‐atomic coupling between the 4*s* and 3*d* orbitals in Cu, due to the reduction of the core electron repulsion [84, 85], thereby reducing the availability of these orbitals for hybridization with external species such as Zn. In contrast, the C‐edge features unsaturated dangling bonds due to the absence of *σ*‐bonded neighbors, resulting in pDOS peaks that are distributed below the Fermi level (*E*_f_), including the energy region where Zn 3*d* orbital exists [86]. These unsaturated edge carbon sites introduce localized C‐2*p* states, which make the C‐edge energetically favorable for chemical interaction with metal species [87, 88]. Accordingly, the orbital overlap between Zn 3*d* states and C‐edge *2p* states provides a plausible electronic basis for strong Zn–C‐edge bonding. This electronic alignment facilitates stronger orbital hybridization between Zn and the C‐edge, making it more favorable than the interaction observed on the Cu(111) surface.

To assess the interfacial behavior of Zn^2+^, we investigated the desolvation process by determining the activation energy (*E*_a_) from temperature‐dependent EIS measurements [89] (Figure S18), based on the Arrhenius relationship. Symmetric cells were constructed using Zn‐deposited Cu electrodes (Zn@Cu) and Zn‐deposited OHNS‐Cu electrodes (Zn@OHNS‐Cu), respectively. The lower *E*_a_ value for Zn@OHNS‐Cu (27.4 kJ mol^−^^1^) compared to Zn@Cu (33.9 kJ mol^−^^1^) indicates enhanced desolvation kinetics. To examine the Zn nucleation behavior on the Cu(111) and C‐edge during the initial stage of electrodeposition, we investigated the thermodynamic stability of Zn aggregation on the Cu(111) surface and C‐edges (Figure 3c and Figure S19). The cohesive energy of Zn (*E*_coh,Zn_, pink‐dashed line) was selected as a reference value for the probability of atomic clustering by the adsorbed Zn atoms. The adsorption energy of a single Zn atom on the pristine Cu(111) (−1.20 eV) is more positive than *E*_coh,Zn_ (−1.27 eV), indicating that the formation of Zn aggregates is thermodynamically preferred to the deposition on the Cu(111) surface. In contrast, the Zn adsorption energy at the C‐edge (−2.18 eV) exhibited a more negative magnitude relative to *E*_coh,Zn_, suggesting that Zn atoms bound to the C‐edge are more thermodynamically stable than their agglomeration. A consistent behavior was observed with the incremental adsorption of Zn atoms on the surface. In addition, the effect of different adsorption configurations on the thermodynamic stability was evaluated. When two or three Zn atoms were adsorbed on the Cu(111), the clustered configuration was more stable than the dispersed configuration. By comparison, the dispersed configuration exhibited high stability at the C‐edge. This is mainly because when Zn adsorbs in a clustered state, each Zn atom shares the same C atom, indicating the orbital hybridization with less C‐edge [90]. Hence, Zn adsorption at the C‐edge energetically favors the dispersed configuration, indicative of a uniform distribution of Zn nuclei without aggregation.

When an additional fourth Zn atom is adsorbed, the adsorption configuration can be classified as either vertical stacking or lateral dispersion (Figure S20). The relative energies of the two configurations are critical in controlling the formation of Zn dendrites [91]. The energy difference between lateral and vertical clusters on the Cu(111) surface is negligible, implying the possibility of Zn dendrite growth on the Cu(111). In contrast, for C‐edges, lateral dispersion of adsorbed Zn atoms is favored over vertical adsorption. This indicates that C‐edges effectively stabilize the lateral adsorption state and prevent dendrite formation along the vertical direction of the GONR. These findings confirm that the C‐edges of GONRs facilitate orbital hybridization with Zn atoms, enabling the formation of an orbital‐hybridizable nanoseed (OHNS) interphase.

To clarify the effect of orbital hybridization on the modulation of Zn nucleation kinetics, we estimated the nucleation overpotential (*η*_nucleation_) [92] during the initial stage of Zn electrodeposition at various current densities (0.5, 1, 5, 20, 50, and 100 mA cm^−2^) (Figure 3d–f). The OHNS‐Cu presented the lower *η*_nucleation_ values than the pristine Cu over all current densities examined herein, indicating that the OHNS interphase can promote the uniform nucleation even under fast‐charging conditions.

This Zn nucleation is followed by the 2D surface diffusion of Zn atoms, resulting in polycrystalline aggregates and mossy Zn networks due to cluster impingement [93]. To achieve homogeneous Zn deposition, it is desirable to restrict the 2D surface diffusion of Zn adatoms [94]. The activation energy barrier for the 2D surface lateral diffusion of Zn adatoms along the C‐edges of the GONR and Cu(111) sites was determined by conducting Nudged elastic band (NEB) calculations (Figure 3g, Figure S21 and Table S5). The Zn atoms adsorbed on the Cu(111) sites exhibited low diffusion energy barriers of 0.05 eV, indicating the facile diffusion in the lateral direction. In contrast, the C‐edge sites exhibited a high diffusion energy barrier (1.30 eV). This could be attributed to the strong affinity between the C‐edge and Zn, surpassing the Zn atoms’ cohesive forces. The chronoamperometry (CA) analysis was conducted to investigate Zn diffusion behavior on the pristine Cu and OHNS‐Cu (Figure S22). The current density of the pristine Cu continuously increased over 500 s, indicating a rampant 2D diffusion and an increase in the effective surface area. In comparison, the OHNS‐Cu reached a steady current density after the short nucleation transient, indicating the predominance of 3D diffusion of Zn [95].

These results revealed that the OHNS interphase (enriched with the C‐edges) enabled the formation of uniform Zn nuclei and effectively restricted the 2D surface diffusion, which is a consequence of the strong orbital hybridization of the C‐edges with the Zn atoms. To verify that this improvement does not simply arise from the presence of carbon layers and oxygen functional groups, we prepared a GO‐coated Cu (GO‐Cu) as a control sample under the same coating protocol. While GO also contains oxygen functionalities, it has a much lower density of C‐edges than the unzipping‐derived GONR framework [51]. Compared with GO‐Cu, OHNS‐Cu exhibited lower nucleation overpotentials (Figure S23) and suppressed 2D surface diffusion (Figure S24). In Zn||Cu cells, OHNS‐Cu further delivered stable cycling with an average CE of 99.5% over 250 cycles, whereas GO‐Cu maintained 99.3% for only 138 cycles (Figure S25). These results indicate that the performance improvement is governed by the electronic interactions between C‐edges and Zn, as evidenced by the Zn–C orbital hybridization in our DFT and pDOS analyses, rather than by the mere presence of carbon coverage or oxygen‐containing functional groups.

### 2.3 Structural Evolution of Zn During Electrodeposition at High Current Rates

**FIGURE 4:**
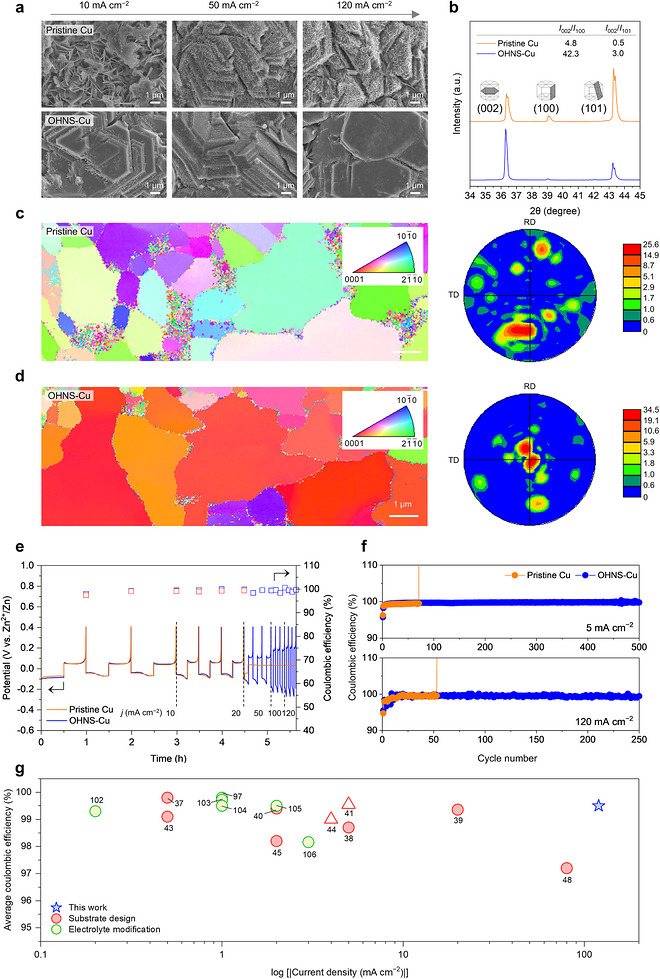
Structural evolution of Zn during the electrodeposition at high current rates. (a) SEM images of pristine Cu (top) and OHNS‐Cu (bottom) after Zn electrodeposition at various current densities (10, 50, and 120 mA cm^−2^) with an areal capacity of 5 mAh cm^−2^. (b) XRD patterns for the crystallographic evolution of Zn electrodeposited on pristine Cu and OHNS‐Cu at a current density of 120 mA cm^−2^ and an areal capacity of 5 mAh cm^−2^. EBSD maps and corresponding (0001) pole figures were performed from the cross‐sectional direction of Zn electrodeposited on (c) pristine Cu and (d) OHNS‐Cu at a current density of 120 mA cm^−2^ and an areal capacity of 5 mAh cm^−2^. (e) Rate performance of Zn||Cu cells (pristine Cu vs. OHNS‐Cu) at current densities (*j*) ranging from 10 to 120 mA cm^−2^ and an areal capacity of 5 mAh cm^−2^. (f) Coulombic efficiency (CE) of Zn plating/stripping in Zn||Cu cells (pristine Cu vs. OHNS‐Cu) at current densities of 5 mA cm^−2^ (top) and 120 mA cm^−2^ (bottom) under an areal capacity of 2 mAh cm^−2^. (g) Comparison of rate performance of Zn||Cu asymmetric cells between this study and previously reported Zn metal electrodes, focusing on current density (*x*‐axis) and average CE (*y*‐axis), in which some references marked with triangle indicate absence of the reported average CE.

The structural evolution of the Zn deposited on the pristine Cu and OHNS‐Cu was investigated at a high areal capacity (5 mAh cm^−2^) as a function of current densities varying from 10 to 120 mA cm^−2^. For the pristine Cu (Figure 4a (top)), the uneven distribution of clustered Zn dendrites was observed, which became more pronounced at the higher current densities. In sharp contrast, the OHNS‐Cu (Figure 4a (bottom)) exhibited the planar Zn deposition with a hexagonal‐close‐packed morphology. In addition, the Zn growth process was examined through SEM (Figure S26) and atomic force microscopy (Figure S27) analyses at different areal capacities under a fixed high current density of 120 mA cm^−2^. As the deposition capacity increased, the pristine Cu exhibited a random morphology that gradually evolved into irregular and loosely packed Zn, whereas the OHNS‐Cu showed uniformly distributed hexagonal nucleation, leading to compact Zn deposits. Furthermore, in situ optical microscopy (OM) was performed to provide time‐resolved visualization of Zn deposition morphology on pristine Cu and OHNS‐Cu current collectors (Figure S28). On pristine Cu, Zn deposition proceeded via non‐uniform nucleation followed by the development of mossy deposits and dendritic features. In contrast, OHNS‐Cu exhibited more homogeneous nucleation and a compact, uniform Zn deposit with effectively suppressed dendritic growth.

The crystallographic orientation of the Zn deposited at a high current density of 120 mA cm^−2^ was systematically examined by XRD patterns and electron backscatter diffraction (EBSD). The Zn(002) crystal plane, which is the most densely packed plane, allows high reversibility compared to the Zn(101) and Zn(100) planes [96]. In the diffraction pattern of Zn deposited on the pristine Cu, the diffraction peak representing the (101) plane was predominantly observed (Figure 4b). For the Zn deposited on the OHNS‐Cu, the strong diffraction peaks on the (002) plane confirmed the (002)‐oriented Zn deposition, demonstrating the preferential orientation under high current density. The orientation anisotropy of deposited Zn was evaluated by analyzing the peak intensity ratios of Zn(002)/Zn(100) (*I*_002_/*I*_100_), and Zn(002)/Zn(101) (*I*_002_/*I*_101_). To elucidate the capacity‐dependent evolution of Zn crystallographic texture under the fast plating condition (120 mA cm^−2^), we conducted a staged (quasi time‐resolved) ex situ XRD analysis after plating Zn on pristine Cu and OHNS‐Cu to areal capacities of 0.5, 1, 3, and 5 mAh cm^−2^ (Figure S29). Across all capacities, Zn deposited on OHNS‐Cu exhibits consistently higher *I*_002_/*I*_100_ and *I*_002_/*I*_101_ values than that on pristine Cu, and these ratios further increase with plated capacity on OHNS‐Cu, indicating progressively strengthened Zn(002)‐textured growth and preferential orientation along the (002) plane [97, 98]. This crystallographic preference was further corroborated by the EBSD maps and the corresponding pole figures. The Zn deposited on the pristine Cu exhibited a random and disordered crystal plane of Zn (Figure 4c). In comparison, the Zn deposited on the OHNS‐Cu exhibited a (002)‐dominated crystal facet of Zn (Figure 4d), indicating a locked crystal orientation [99, 100]. This (002)‐oriented Zn growth is attributed to the zincophilic C‐edge sites, which strongly adsorb Zn atoms, thereby lowering the surface energy of the Zn(002) facet and promoting its growth [101].

As a supplementary experiment to explore the high‐current performance of the OHNS‐Cu in aqueous Zn batteries, the rate performance of Zn||Cu asymmetric cells was examined as a function of current densities ranging from 10 to 120 mA cm^−2^ at a fixed areal capacity of 5 mAh cm^−2^ (Figure 4e). For the pristine Cu, the cell failed to operate at a current density of 50 mA cm^−2^ because of the severe growth of Zn dendrites. In comparison, the OHNS‐Cu exhibited the reversible Zn plating/stripping even at higher current densities of up to 120 mA cm^−2^. Furthermore, the OHNS‐Cu cells achieved the mitigated voltage hysteresis and superior cycling stability with a high average CE of 99.7% at a current density of 5 mA cm^−2^ and, more notably, 99.5% even at a higher current density of 120 mA cm^−2^ (Figure 4f, Figures S30 and S31). This fast and reversible Zn plating/stripping of the OHNS‐Cu showed improved performance those of previous studies on Zn metal batteries [37, 38, 39, 40, 41, 43, 44, 45, 48, 97, 102, 103, 104, 105, 106] (Figure 4g and Table S6). At a low current density, the OHNS‐Cu exhibited enhanced Zn reversibility, confirmed by electrochemical analysis (Figures S32 and S33), uniform Zn morphology (Figure S34), and suppression of dendrite growth (Figure S35).

### 2.4 Suppressing H_2_ Evolution and Zn Corrosion

**FIGURE 5:**
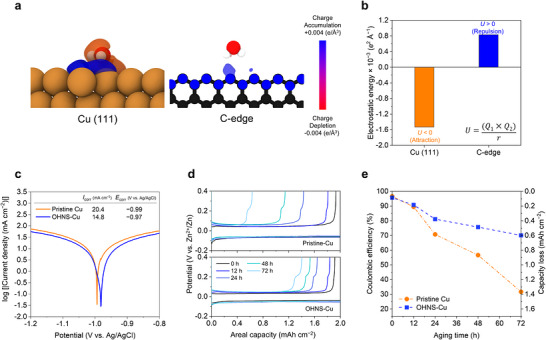
Suppressing H_2_ evolution and Zn corrosion. (a) Charge density difference of H_2_O and adsorption sites (Cu(111) vs. C‐edge) with an isosurface level of 0.004 e Å^−3^. (b) Electrostatic interaction energies of Cu(111) and C‐edge with H_2_O. (*U*: electrostatic energy, *Q*_1_: Bader charge for H in H_2_O, *Q*_2_: Bader charge for C in the C‐edge or Cu in Cu(111), and *r*: perpendicular distance) (c) Tafel plot after Zn electrodeposition of 2 mAh cm^−2^ (pristine Cu vs. OHNS‐Cu). (d) Charge/discharge profiles of Zn||Cu cells as a function of aging time (pristine Cu (top) vs. OHNS‐Cu (bottom)), in which current collectors were initially electroplated with 2 mAh cm^−2^ of Zn and then aged for different durations (0, 12, 24, 48, and 72 h). Subsequently, the Zn‐deposited Cu electrodes were stripped to 0.4 V (vs. Zn^2+^/Zn) at a current density of 5 mA cm^−2^. (e) CE and capacity loss as a function of aging time (pristine Cu vs. OHNS‐Cu).

According to the Pourbaix diagram, the reduction of Zn^2+^ on anodes competes with the H_2_ evolution reaction via water hydrolysis [107]. A significant portion of the transferred electrons to the Zn anode are consumed for the proton reduction instead of the Zn deposition, which inevitably degrades the efficiency of Zn plating/stripping. Therefore, inhibiting the H_2_ evolution at Zn anodes is necessary to improve the cycle reversibility [108]. We performed the DFT calculations to investigate the occurrence of H_2_ evolution during the initial Zn nucleation stage. This allowed us to determine the charge distribution on the surface following the adsorption of an H_2_O molecule on the Cu(111) or C‐edge sites (Figure 5a). A significant difference in the charge density between the H_2_O molecule and the adsorption sites is known to reveal a strong electrostatic attraction, indicating the H_2_ evolution via the proton reduction [109]. Compared to the Cu(111), which exhibited a charge transfer to the H_2_O molecule, the C‐edge demonstrated a small difference in the charge density, thereby suppressing the H_2_ evolution and byproduct formation.

This result was verified by quantitatively calculating the electrostatic energy [110] between the H_2_O molecule and the adsorption sites. The C‐edge surface exhibited a repulsive interaction (+0.833 × 10^−3^ e^2^ Å^−1^) toward H_2_O, whereas the Cu(111) showed an attractive interaction (−1.532 × 10^−3^ e^2^ Å^−1^) (Figure 5b and Table S7). The hydrophobic feature of the OHNS‐Cu was experimentally verified by measuring its surface contact angle with water (Figure S36). Due to the nonpolar *sp* ^2^‐hybridized carbons in the GONR, the OHNS‐Cu had a higher contact angle of 82.8° compared to that of the pristine Cu (71.4°). The water‐repellency of the OHNS‐Cu contributes to suppressing the water‐induced interfacial side reactions. In addition, to investigate HER suppression during the Zn electrodeposition process, we performed in situ differential electrochemical mass spectrometry (DEMS). The DEMS analysis confirmed a 79.7% reduction in cumulative H_2_ evolution for the OHNS‐Cu compared to the pristine Cu at a cathodic scan rate of 0.1 mV s^−1^ (Figure S37). Notably, under the fast‐plating condition at a high current density of 120 mA cm^−2^, in situ DEMS showed a lower H_2_ signal on OHNS‐Cu than on pristine Cu (Figure S38), confirming effective suppression of H_2_ evolution across distinct electrochemical regimes. Meanwhile, the OHNS‐Cu exhibited a lower contact angle (58.6°) with the aqueous electrolyte compared to the pristine Cu (62.9°), indicating enhanced electrolyte wettability (Figure S39). This behavior is attributed to the high Zn adsorption energy of OHNS‐Cu arising from orbital hybridization at the C‐edge sites. Consequently, the OHNS interphase mitigates potential mass transport limitations while simultaneously suppressing water‐induced side reactions. Linear sweep voltammetry (LSV) was performed after electrodepositing 2 mAh cm^−2^ of Zn on pristine Cu and OHNS‐Cu to evaluate HER activity after Zn plating (Figure S40). Zn@OHNS‐Cu required a more negative potential (−0.16 V) than Zn@Cu (−0.10 V) to reach a cathodic current density of −10 mA cm^−2^, indicating a larger HER overpotential and thus more effective HER suppression. This behavior can be attributed to the OHNS interphase regulating Zn deposition toward a more Zn(002)‐textured and compact morphology, thereby reducing electrochemical activity toward HER [111].

Based on this understanding of the surface characteristics of the electrodes, a Tafel plot analysis was conducted to investigate their corrosion behavior in aqueous electrolytes, in which 2 mAh cm^−2^ of Zn was electrodeposited on the pristine Cu and OHNS‐Cu (Figure 5c). The OHNS‐Cu exhibited a higher corrosion potential and a reduced corrosion current compared to the pristine Cu, indicating an alleviated corrosion reaction activity. The Zn metal undergoes degradation during aging and cycling in aqueous electrolytes due to interfacial side reactions with water molecules, which promotes the irreversible conversion of Zn into corrosive byproducts [112, 113].

Capacity loss was measured as a function of aging time to quantify the extent of Zn corrosion (Figure 5d,e) [114]. The current collectors were initially electroplated with 2 mAh cm^−2^ of Zn. The cells were then aged for different durations (0, 12, 24, 48, and 72 h). Subsequently, the Zn‐deposited Cu electrodes were stripped to 0.4 V (vs. Zn^2+^/Zn). Compared to the pristine Cu, the OHNS‐Cu had a higher Coulombic efficiency and lower capacity loss throughout the aging period. In addition, the OHNS‐Cu exhibited a mitigated degradation in the aging capability over the elapsed time. This superior performance of the OHNS‐Cu is attributed to the synergistic effects of suppression of H_2_ evolution (Figures S37 and S40), enhancement of corrosion resistance (Figure S41), and mitigation of dead Zn formation (Figure S35), achieved by the (002)‐textured Zn deposits [111, 115].

### 2.5 Fast Rechargeability of Energy‐Dense Aqueous Zn Full Cells

To investigate the effect of the OHNS interphase on the cell performance, aqueous Zn full cells were fabricated with Zn@OHNS‐Cu as an anode and CaV_6_O_16_·3H_2_O (CVO) [116] as a cathode. The galvanostatic charge/discharge measurement was conducted to examine the cycling stability of the cells at a high current density of 5 A g^−1^ (Figure S42). The cell with the Zn@Cu anode (control sample) exhibited rapid capacity degradation due to dendrite formation and interfacial side reactions. In comparison, the Zn@OHNS‐Cu anode enabled the full cell to deliver a stable capacity retention of 91.8% after 3000 cycles with an average CE above 99.6%. The OHNS‐Cu cell also demonstrated long‐term cycling stability at low current density (0.3 A g^−1^) (Figure S43).

**FIGURE 6:**
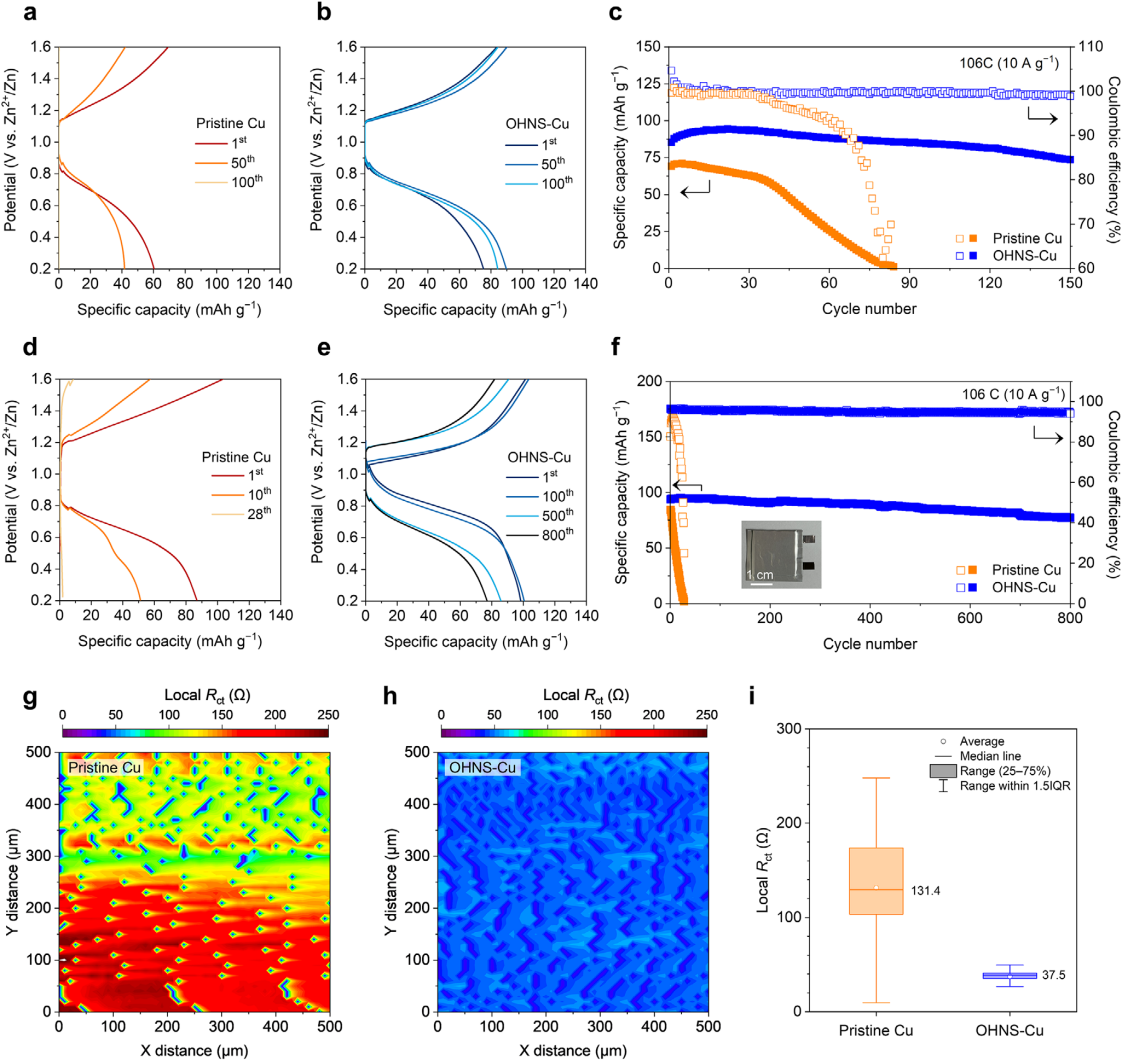
Fast rechargeability of energy‐dense aqueous Zn full cells. Charge/discharge profiles of anode‐free full cells at a current density of 10 A g^−1^: (a) pristine Cu and (b) OHNS‐Cu. (c) Cycling performance of anode‐free full cells. Charge/discharge profiles of anode‐free pouch cells at a current density of 10 A g^−1^: (d) pristine Cu and (e) OHNS‐Cu. (f) Cycling performance of anode‐free pouch cells. The inset is a photograph of an anode‐free pouch cell. LEIS area scans of (g) pristine Cu after 84 cycles and (h) OHNS‐Cu after 150 cycles in anode‐free full cells. (i) Box chart of LEIS area scans. Dots represent the average values, while the boxes denote the 25%–75% range. The horizontal lines in the boxes indicate the median values, and the whiskers extend to the maximum and minimum values, excluding outliers.

We exploited the anode‐free cell configuration to increase the cell energy density by assembling the OHNS‐Cu (without Zn) anode and pre‐zincificated CVO cathode (Zn*_x_* CVO). The resulting anode‐free full cells exhibited a maximum energy/power density of 140.6 Wh kg^−1^/4138.1 W kg^−1^ based on the total mass of electrodes. The charge/discharge behavior of the anode‐free full cells was investigated as a function of current densities varying from 0.1 to 10 A g^−1^ (Figure S44). Compared to the pristine Cu anode, the OHNS‐Cu anode exhibited higher charge/discharge capacities in the anode‐free full cell over the whole current densities. At a fast charge/discharge current density of 106C, corresponding to 34 s, the anode‐free full cell with the pristine Cu exhibited severe capacity decay due to the uncontrolled Zn dendrites and interfacial side reactions. In contrast, the anode‐free full cell with the OHNS‐Cu anode exhibited exceptional cycling stability (capacity retention = 86.3% after 150 cycles) at the fast current density of 106C (Figure 6a–c). Moreover, the OHNS‐Cu cell maintained long‐term cycling stability even at a low current density (Figure S45). To demonstrate the practical applicability of OHNS‐Cu, we fabricated anode‐free pouch cells. Under a fast charge/discharge rate of 106C, the pouch cell with OHNS‐Cu exhibited superior capacity retention (82.2% after 800 cycles), whereas the pristine Cu pouch cell rapidly failed (2.3% after 28 cycles) (Figure 6d–f). This improved cycling stability is further corroborated by comparison with previously reported anode‐free Zn systems [19, 26, 27, 28, 29, 31, 42, 62, 64, 117, 118, 119, 120, 121, 122] (Figure S46 and Table S2).

This beneficial effect of the OHNS‐Cu anode on the fast rechargeability and stable cycle life was further elucidated by conducting local electrochemical impedance spectroscopy (LEIS) [123] analysis (Figure 6g–i). Compared to the cycled pristine Cu anode (after 84 cycles), demonstrating an uneven ionic topology with high local charge transfer resistance (*R*_ct_) (average = 131.4 Ω, standard deviation = 53.8 Ω), the cycled OHNS‐Cu anode (after 150 cycles) exhibited a uniform ionic topology with low local *R*_ct_ (average = 37.5 Ω, standard deviation = 6.6 Ω). This result was further corroborated by post‐mortem SEM and XRD analyses of pristine Cu and OHNS‐Cu current collectors obtained from anode‐free full cells after cycling at 10 A g^−1^. Post‐cycling SEM images (Figure S47) show that pristine Cu develops a rough and heterogeneous surface with dendritic features, whereas OHNS‐Cu maintains a more uniform and smooth morphology with minimized inactive Zn accumulation. In addition, post‐cycling XRD (Figure S48) reveals clear diffraction peaks of Zn_4_SO_4_(OH)_6_·4H_2_O (ZHS) on pristine Cu, indicating pronounced formation of corrosion byproducts, while ZHS formation is strongly suppressed on OHNS‐Cu. Post‐cycling XPS was performed to evaluate the chemical and structural stability of the GONRs in the OHNS interphase after prolonged cycling (Figure S49). The cycled OHNS‐Cu exhibited C 1*s* features comparable to those of pristine OHNS‐Cu, with no noticeable spectral changes, no emergence of new components, and no spectral signatures indicative of interphase degradation. These results indicate that the graphitic framework and surface chemical states of the GONR‐based interphases on OHNS‐Cu remain stable during cycling. Together with the post‐cycling morphological/phase analyses described above, such interphase stability underscores the important role of the OHNS interphase in enabling fast and reversible Zn plating/stripping in aqueous Zn batteries.

## 3 Conclusion

We demonstrated that the OHNS interphase based on the GONR enables the realization of one‐minute rechargeable, energy‐dense anode‐free aqueous Zn batteries. The C‐edges prevalent in the GONR allow for the heteroatom orbital hybridization with 3*d* orbitals of Zn, enhancing the Zn nucleation kinetics and simultaneously retarding the 2D surface diffusion of adsorbed Zn. Consequently, the homogeneous, dense, and (002)‐oriented growth of Zn was obtained, facilitating the reversible Zn plating/stripping with high Coulombic efficiency (∼ 99.5%) at a high current density of 120 mA cm^−2^ while suppressing water‐triggered interfacial side reactions in aqueous electrolytes. Benefiting from these advantageous effects, the OHNS interphase achieved a stable capacity retention of 91.8% after 3000 cycles with an average CE above 99.6% in the Zn full cell. Notably, the anode‐free full cells with the OHNS‐Cu exhibited a maximum energy/power density of 140.6 Wh kg^−1^/4138.1 W kg^−1^ based on the total mass of electrodes. Moreover, anode‐free pouch cells exhibited stable capacity retention of 82.2% after 800 cycles at a fast charge/discharge current density of 106C (equivalent to a time of 34 s), which exceeded those of previously reported aqueous Zn batteries. This nanoseed interphase strategy is expected to serve as a versatile platform for emerging metal batteries (e.g., sodium, calcium, potassium, and aluminum), which suffer from chemical instability and limited electrochemical reliability at the electrode–electrolyte interface, especially under high current operating conditions. Beyond its practical contributions, the underlying mechanism—orbital hybridization between heteroatoms facilitating (002)‐oriented Zn growth at high electrodeposition current densities—may have broader implications across various research domains involving reversible metal plating and stripping.

## 4 Experimental Section/Methods

### 4.1 Materials

ZnSO_4_·7H_2_O, Ca(CH_3_COO)_2_, and V_2_O_5_ were purchased from Junsei, Sigma–Aldrich and Pechiney, respectively. Deionized water (DI) was utilized to prepare aqueous electrolytes with a water purification system (Direct Q3, Millipore). Zn foils (thickness = 250 µm), Cu foils (thickness = 18 µm), SUS304 foils (thickness = 80 µm), and glass fiber separators (thickness = 180 µm) were purchased from Alfa Aesar, Welcos, NanoNC, and Whatman, respectively. CVO cathode materials were synthesized accordingly with a previously reported microwave‐assisted hydrothermal method [116]. The CVO cathode was prepared by casting a slurry mixture of CVO, carbon black, and polyvinylidene fluoride in a ratio of 7:2:1 (w/w/w) in *N*‐methyl‐2‐pyrrolidone (NMP) onto a SUS304 substrate. Subsequently, the prepared cathode was vacuum‐dried at 60°C for 12 h. The mass loading of cathode active materials ranged from 1.5 to 2.5 mg cm^−2^.

### 4.2 Fabrication of OHNS‐Cu

The detailed preparation of the oxidized MWNTs and GONR has been reported by previous reports [79, 80]. Briefly, 4 g of MWNTs were dispersed in 200 mL of concentrated sulfuric acid, to which KMnO_4_ was added to initiate the unzipping process. The degree of oxidation was controlled by differing the reaction time from 1, 5, and 32 h. Fully unzipped GONRs were obtained after 32 h of oxidation. Finally, 350 mL of DI water was added to the mixture, followed by 80 mL of hydrogen peroxide, to terminate the reaction. The resulting products were collected and rinsed with DI water via vacuum filtration until a neutral pH was achieved. The coating ink solutions were prepared by dispersing the GONR in acetone to reach a concentration of 2.5 mg mL^−1^. A slot‐die coater was utilized to deposit GONR on Cu foils. The Cu foil was laid on the stage, ensuring no creases were formed. The speed of the stage was set as 5 mm s^−1^, and the ink was injected into the coater by a syringe pump at a rate of 1.1 mL min^−1^. The GONR‐deposited Cu foil was finally dried for 1 min at room temperature. Finally, the GONR‐deposited Cu foil was stored in a vacuum state until usage.

### 4.3 Fabrication of GO‐Cu

GO was synthesized via the oxidation process in previous works [124]. Specifically, 2 g of graphite powder was dispersed in 150 mL of concentrated sulfuric acid, followed by the addition of 7 g of KMnO_4_. After 5 h of reaction, 200 mL of DI water and 100 mL of H_2_O_2_ were sequentially added to terminate the reaction. The resulting GO was repeatedly washed with DI water by vacuum filtration, and subsequently freeze‐dried. The obtained GO powder was dispersed in acetone (2.5 mg mL^−1^) to prepare the coating ink. Slot‐die coating, drying, and vacuum storage were conducted using the same procedure as described for the fabrication of OHNS‐Cu.

### 4.4 Characterization

The surface morphologies of the samples were examined by field emission secondary electron microscopy (FE‐SEM, S‐4800, Hitachi), high‐resolution transmission electron microscopy (HR‐TEM, JEM‐2100Plus, JEOL), and energy‐dispersive X‐ray spectroscopy (EDS, JSM 6400, JEOL) detector at an acceleration voltage of 200 kV. Adsorption tests were conducted by dispersing 30 mg of each carbon material in a 300 ppm Zn(NO_3_)_2_ aqueous solution. After 5 h, the supernatant was collected, and the residual Zn^2+^ concentration was quantified by inductively coupled plasma optical emission spectroscopy (ICP‐OES, Agilent 5110, Agilent). The adsorbed amount of Zn^2+^ was calculated from the concentration change. Atomic force microscopy (AFM, NX‐10, Park Systems) was utilized to investigate the Zn nucleation and growth mechanism by using an AFM probe (Park Systems, *f*_0_ = 330 kHz, *k* = 42 N m^−1^). Crystallographic analysis was carried out via XRD (SmartLab, Rigaku) with Cu Kα radiation at 45 kV and 200 mA. The contact angles between the current collectors and water were measured at room temperature utilizing a contact angle analyzer (DSA100, KRUSS). The EBSD (Quattro S, FEI FEI) measurement was performed on the cross‐section of the Zn‐electrodeposited current collectors, in which the electrodeposition was conducted at a current density of 120 mA cm^−2^ and a capacity of 5 mAh cm^−2^. Subsequently, the samples were then subjected to argon ion milling (IB‐19520CCP, JEOL) under 6 kV conditions to obtain a cross‐sectional specimen.

### 4.5 Fabrication of Aqueous Zn Cells and Their Electrochemical Characterization

The Zn||Cu cells (CR2032 coin) were fabricated by assembling the Zn foil, Cu foil, glass fiber separator, and aqueous electrolyte (2 m ZnSO_4_ in H_2_O, 50 µL per cell). Symmetric coin cells (CR2032) were assembled using two identical electrodes, with Zn pre‐deposited onto either pristine Cu (Zn@Cu) or OHNS‐Cu (Zn@OHNS‐Cu). Zn pre‐deposition was performed at a current density of 1 mA cm^−^^2^ to an areal capacity of 5 mAh cm^−^^2^. The EIS was carried out in the frequency range from 10^−1^ to 10^6^ Hz with an applied voltage of 10 mV. In situ OM analysis was performed in Zn||Cu cells using pristine Cu or OHNS‐Cu as the working electrode and Zn foil as the counter electrode during CA tests at −1 V (vs. Zn^2+^/Zn). The in situ DEMS measurement was conducted to monitor hydrogen gas evolution occurring during the cathodic scan at 0.1 mV s^−1^ and during Zn plating and stripping at 120 mA cm^−2^ with an areal capacity of 2 mAh cm^−2^. Gas evolution in the cells was recorded at 5 min intervals. For LSV measurements, Zn was electrodeposited onto pristine Cu and OHNS‐Cu at 1 mA cm^−2^ to an areal capacity of 2 mAh cm^−2^. Subsequently, Zn deposited Cu electrodes were assembled into Zn||Zn deposited Cu cells and evaluated by LSV at a scan rate of 1 mV s^−1^. The CA measurements and Tafel plot were conducted with a three‐electrode cell at room temperature. The working electrode was a Cu current collector, the counter electrode was a Zn foil, and the reference electrode was an Ag/AgCl electrode (filled with a saturated KCl electrolyte). After electrodepositing Zn on both the pristine Cu and OHNS‐Cu at a current density of 1 mA cm^−2^ and an areal capacity of 2 mAh cm^−2^, a Tafel plot analysis was performed. In addition, the CE measurements were conducted with the Zn||Cu cells. For the corrosion test, the CE was calculated based on Equation (1).

(1) $$\mathrm{CE}\;\left(\%\right)=t_{s}/t_{p}\times 100$$

where *t_p_* and *t_s_* mean the time spent in plating Zn and the time spent in stripping Zn, respectively.

The capacity loss was determined with Equation (2) [114].

(2) $$\mathrm{Capacity}\;\mathrm{loss}=\frac{t_{p}-t_{s}}{t_{p}}\cdot \mathrm{anode}\;\mathrm{loading}$$

The full cells (CR2032 coin) were fabricated by assembling the current collector with electrodeposited Zn (5 mAh cm^−2^) as the anode, CVO cathode, glass fiber separator, and aqueous electrolyte (3.4 m ZnSO_4_ in H_2_O). The N/P ratio of the full cells was calculated based on Equation (3) as the ratio of the areal capacity of Zn electrodeposited on Cu to that of the cathode [125].

(3) $$N/P\;\mathrm{ratio}\;=\frac{\mathrm{Areal}\;\mathrm{capacity}\;\mathrm{of}\;\mathrm{negative}\;\mathrm{electrode}\;\;\left(\mathrm{mAh}\;cm^{-2}\right)}{\mathrm{Areal}\;\mathrm{capacity}\;\mathrm{of}\;\mathrm{positive}\;\mathrm{electrode}\;\left(\mathrm{mAh}\;cm^{-2}\right)}$$

To prepare the Zn*_x_* CVO cathode, the CVO electrode underwent pre‐zincification utilizing a Zn||CVO cell. Anode‐free full cells (CR2032 coin and 2 × 2 cm^2^ pouch type) were assembled using Zn*_x_* CVO cathodes, bare current collectors (without Zn), a glass fiber separator, and 3.4 m ZnSO_4_ aqueous electrolyte (50 µL for coin cells and 1 mL for pouch cells). The cells were charged to 1.6 V before cycling. The galvanostatic charge/discharge measurements were conducted with a cycle tester (PNE Solution Co., Ltd, Korea) at ambient temperature. The LEIS area scans were carried out with a fixed frequency of 2 Hz utilizing a scanning probe workstation (M470, Biologic).

### 4.6 First‐Principles Calculations

All calculations were carried out with first‐principles DFT as implemented in the Vienna Ab‐initio Simulation Package (VASP) [126]. The plane waves near the core region were replaced with pseudo‐potentials utilizing a projector augmented wave (PAW) [127] method to describe the interaction among core electrons. The generalized gradient approximation (GGA) with the revised Perdew–Burke–Ernzerhof (RPBE) exchange‐correlation functional [128] was utilized. The Kohn–Sham equation was expanded with a plane wave basis set with a cut‐off energy of 520 eV, and k‐points were sampled with a 5 × 5 × 1 Monkhorst–Pack mesh. We tested computational convergence within 10^−5^ eV and 0.02 eV Å^−1^ for energy and force, respectively. A vacuum space of 15 Å was inserted perpendicular to the surface models to have little interaction with its images. We considered the van der Waals interactions for the interactions between adsorbate and adsorbate employing the DFT‐D3 method [129].

Since the diameter of MWNT is about 15‐25 nm, which can neglect the curvature effect, such as adsorption behavior [130], the GONR models were approximated to a single graphene model (Figure S13). For GONR, six systems were calculated as slab models: basal plane of GONR, edge of GONR, edge of GONR with oxygen‐containing functional groups (‐O‐, ‐OH, and ‐COOH) bonded on the edge, and current collector Cu. Cu bulk is a face‐centered cubic structure, so our calculations selected the most thermodynamically stable Cu(111) surface. We defined the adsorption energy of Zn atom(s) on the surfaces as Equation (4).

(4) $$E_{ads}=\frac{1}{n}\left(E_{system/Zn}\;-\;E_{system}-\;nE_{Zn}\right)$$

where *n*, *E*_system/Zn_, *E*_system_, and *E*_Zn_ mean the number of Zn atoms adsorbed, the DFT energy of the system with adsorbed Zn, the system, and the gas phase of Zn atom, respectively.

## Author Contributions

W.Y.K. and S.Y.L. conceived the study. W.Y.K., A.S., O.K., and S.H. designed the project. A.S., W.Y.K., and H.I.K. performed the experimental characterization and evaluation. O.K. and J.Y.K. fabricated and characterized OHNS‐Cu. S.H. and S.J.H. performed the theoretical calculations. H.S.K. and S.H.K. assisted with the data analysis. K.S.O. assisted in analyzing the crystallographic orientation of Zn. X.L. and S.P. contributed to the synthesis of CVO materials. J.H.H. and S.J.K. conducted the DEMS. B.H., D.W.K., and S.Y.L. supervised the overall project. All authors contributed to finalizing the manuscript.

## Funding

This work was supported by the National Research Foundation of Korea (NRF) funded by the Ministry of Science and ICT (RS‐2024‐00344021, RS‐2023‐00261543, RS‐2024‐00407015, RS‐2025‐14852975 and RS‐2022‐NR068232) and the Ministry of Education (RS‐2024‐00413185). This work was also supported by Korea Institute for Advancement of Technology (KIAT) grant funded by the Korea Government (MOTIE) (RS‐2024‐00420590, HRD Program for Industrial Innovation).

## Conflicts of Interest

The authors declare no conflicts of interest.

## Supporting information




**Supporting File 1**: adma73553‐sup‐0001‐SuppMat.docx.

**Supporting File 2**: adma73553‐sup‐0002‐VideoS1.avi.

## Data Availability

The data that support the findings of this study are available in the Supporting Information of this article.
