# Supplementary material for: Orbital‐Hybridizable Nanoseed Interphase Enables One‐Minute Rechargeable, Energy‐Dense Anode‐Free Aqueous Zinc Batteries
Source: Adv Mater. 2026 May 29;38(37):e73553. doi: 10.1002/adma.73553 (PMC13327178; doi:10.1002/adma.73553)
Supplement: Supplementary file 1 — Supporting File 1: adma73553‐sup‐0001‐SuppMat.docx. [file ADMA-38-e73553-s002.docx]

Supporting Information

Orbital-Hybridizable Nanoseed Interphase Enables One-Minute Rechargeable, Energy-Dense Anode-Free Aqueous Zinc Batteries

Won-Yeong Kim, Ahyeon Son, Ohchan Kwon, Suseong Hyun, Sung Jun Hong, Hong-I Kim, Ju Yeon Kim, Hyunseo Kang, Seung-Hyeok Kim, Xu Liu, Kyeong-Seok Oh, Jee Ho Ha, Seok Ju Kang, Stefano Passerini, Byungchan Han*, Dae Woo Kim*, and Sang-Young Lee*

**Figure S1**. TEM images of pristine (a) MWNT and (b) GONR synthesized via oxidative unzipping. KMnO_4_ initially attacks the carbon-carbon bonds of the MWNTs, forming an intermediate manganate species at the nanotube sidewalls. The subsequent reaction involves the cleavage of these intermediates as diketones, causing defects in the graphitic structure. These defects act as initiating points for further oxidation, leading to the unzipping process propagation along the nanotubes’ length. The unzipping process results in the formation of graphene-like ribbon structures along with oxidized carbon species. Dense carbon dangling bonds arise with the development of oxygen-containing functional groups at the unzipped edges of the graphene planes.

**Figure S2**. (a) C 1*s* XPS spectra of MWNTs and GONR, and (b) ratios of oxygen-containing functional groups. The C 1*s* spectra of the samples were deconvoluted into five characteristic peaks at 284.5, 285.5, 286.5, 288.5, and 290.5 eV, corresponding to the *sp*^2^ C-C, C-OH, C-O-C, C=O, and π→π* (shake-up satellite peak), respectively. The measured C/O ratios of the samples, arranged according to oxidation time, were 12.1, 6.6, 3.7, and 2.7, indicating a proportionality between the oxygen group content and the oxidation degree.

**Figure S3**. Electrical conductivities of MWNTs and GONR, determined via four-probe method on foils prepared by depositing carbon materials onto porous polymeric substrates by vacuum filtration. The conductivity of GONR was measured as 0.01 S cm^−1^, which is four orders of magnitude lower than that of pristine MWCNTs, with a conductivity of 16.30 S cm^−1^. This decrease in conductivity is attributed to a decrease in the number of electrically conductive sp^2^ carbons.

**Figure S4**. SEM images of planar GONR scaffolds after extrusion from slot-die coater. The scaffolds were obtained by directly collecting the extruded GONRs in liquid nitrogen at the slot-die coater lip.

**Figure S5**. Photographs of MWNT-coated Cu foil prepared using acetone-based inks, showing non-uniform and discontinuous coating under identical slot-die conditions.

**Figure S6**. Photographs of GONR-Cu foils with (a) acetone-based and (b) water-based inks. (c,d) Corresponding low and high magnification surface SEM images. The white arrow depicts surface imperfections attributed to the corrosion of the Cu layer. The Cu was oxidized by aqueous GONR dispersions, which may be caused by the residual acid (even after purification), or the transfer of oxygen-containing functional groups from GONR. We note that the dark and light regions observed in the photographic image of the aqueous processing solution are attributable to ink flow and aggregation, arising from incomplete drying associated with the low volatility of the solvent.

**Figure S7**. (a) Survey of polarity and boiling point (volatility) of commercially available processing solvents for GONR ink. (b) Dispersibility of MWNT and GONR in various solvents (dispersion concentration: 1 mg mL^−1^). The increased functionalization of GONR enables stable dispersion across diverse solvents, whereas MWNTs exhibit poor dispersibility under identical conditions. (c) Photographs of GONR-Cu foils prepared with ethanol- and IPA-based inks.

The blue region in **Figure S7a** denotes solvents with normalized polarity (E_T_^N^) values above 0.3, in which GONRs form stable dispersions. The yellow region indicates high-volatility solvents with boiling points below 65 °C at 1 atm. Solvents suitable for stable dispersion are marked by filled circles. Two key parameters govern solvent suitability: E_T_^N^ and volatility. Solvents with E_T_^N^ values above 0.3 enabled stable GONR dispersion; however, excessively high polarity (e.g., water, E_T_^N^ = 1.00) induced surface oxidation of Cu (**Figure S6b**), while nonpolar solvents (e.g., hexane, E_T_^N^ = 0.01) failed to disperse GONRs (**Figure S7b**). Volatility determines drying behavior: solvents with high boiling points (e.g., NMP, b.p. = 202 °C) are prone to incomplete removal and energy-intensive drying[1], whereas moderately volatile solvents (e.g., ethanol, b.p. = 78.3 °C; IPA, b.p. = 82.2 °C) cause local evaporation gradients and non-uniform coatings (**Figure S7c**). By contrast, acetone combines appropriate polarity (E_T_^N^ = 0.36) with high volatility (b.p. = 56.1 °C), allowing stable GONR dispersion and rapid, complete removal during drying, thereby producing reproducible and uniform coatings (**Figure 2f** and **Video S1**).

**Figure S8**. Cross-sectional SEM-EDS images of GONR-deposited Cu foil.

**Figure S9**. SEM images of the (a) 1.0 GONR-Cu, (b) 2.5 GONR-Cu and (c) 5.0 GONR-Cu. Blue shaded regions indicate the exposed Cu foil.

To evaluate the influence of coating solution concentration on the morphology of the GONR layer, Cu substrates were coated using solutions with concentrations of 1.0, 2.5, and 5.0 mg mL^−1^. These samples are referred to as *x* GONR-Cu, with *x* denoting the GONR concentration. SEM characterization revealed that at 1.0 mg mL^−1^, the GONR failed to form a continuous film, leaving portions of the Cu substrate exposed. In contrast, at concentrations of 2.5 mg mL^−1^ and above, the coatings were uniform and continuous, fully covering the underlying Cu surface.

**Figure S10**. (a) Comparison of nucleation overpotentials on pristine Cu and GONR-coated Cu substrates, denoted as *x* GONR-Cu, where *x* represents the GONR concentration (1.0, 2.5, and 5.0 mg mL^−1^) in the coating solution. (b) CE of Zn plating/stripping in Zn||Cu cells (pristine Cu, 1.0, 2.5, and 5.0 GONR-Cu) at a current density of 5 mA cm^−2^ under an areal capacity of 5 mAh cm^−2^.

The electrochemical behavior of the GONR-coated Cu substrates was evaluated as a function of coating solution concentration. The 1.0 GONR-Cu sample, with a non-uniform coating, showed a high nucleation overpotential (64.9 mV), comparable to that of pristine Cu (76.0 mV) (**Figure S10a**). This is attributed to the insufficient continuity of the interfacial layer and the limited availability of zincophilic C-edge sites. In contrast, the 5.0 GONR-Cu sample exhibited a lower nucleation overpotential (32.5 mV) but suffered from low CE and poor cycling stability (**Figure S10b**). Given the intrinsically low electronic conductivity of GONR (**Figure S3**), increasing the coating thickness proportionally increased the vertical electronic resistance across the interfacial layer, as described by the following **Equation S1** [2].

$R=\frac{\rho\cdot L}{A}$ (1)

where 𝑅 is resistance, 𝜌 the resistivity, 𝐿 the thickness, and 𝐴 the cross-sectional area. A thicker GONR layer therefore elevates the resistance along the electron transport pathway, thereby impeding uniform electron transfer during Zn plating/stripping and compromising electrochemical reversibility. Considering both coating uniformity (**Figure S9**) and electrochemical performance (**Figure S10**), the 2.5 GONR-Cu sample was determined to be optimal. Slot-die coating at this concentration yielded a film thickness of ~ 80 nm (**Figure 2h**), which was adopted as the final OHNS interphase.

**Figure S11.** AFM images showing the roughness of: (a) pristine Cu foil, and foils coated with oxidized MWNTs for (b) 1 h, (c) 5 h, and (d) GONR utilizing the slot-die coater. The root mean square roughness (*R*_q_) of the GONR layer exhibits the lowest value (14 nm) among the coated samples, attributed to the denser packing of the unzipped graphene sheets.

 **Figure S12.** (a) N_2_ adsorption-desorption isotherms (at 77 K) of the MWNTs and GONR and (b) their calculated surface areas. *S*_micro_, *S*_BET_ and *S*_external_ represent micropore surface area, Brunauer–Emmett–Teller (BET) surface area, and external surface area, respectively. This analysis reveals a reduction in the *S*_external_ with increased oxidation time, which is consistent with the SEM images. Only the GONR exhibits microporosity among the tested samples, as indicated by the Type I isotherm and the high *S*_micro_. These findings suggest that the functionality and unzipped structure of GONR facilitate higher levels of microdomain assembly, while the relative rigidity of the zipped MWNTs in the 1 h and 5 h samples hinders further ordering. The adsorption results were obtained from the dried powders of the carbon species.


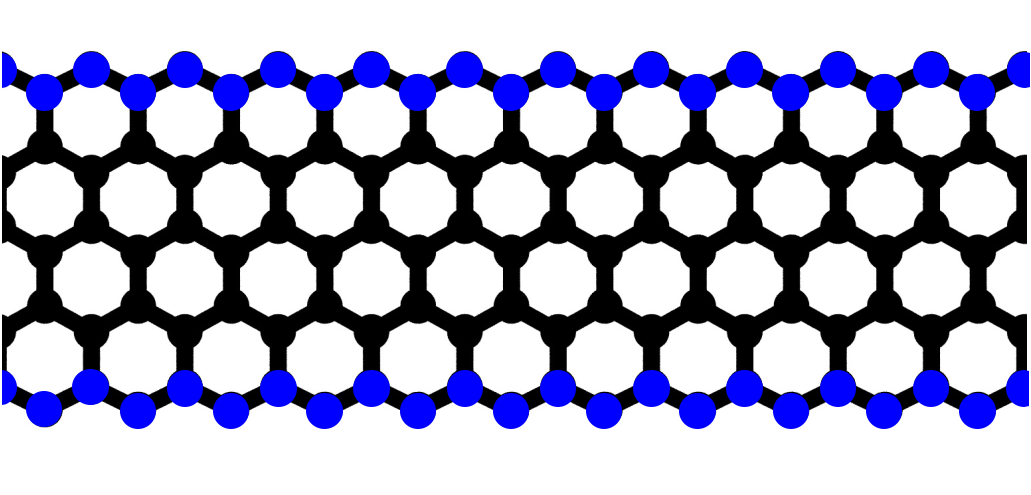


**Figure S13.** Atomistic model of graphene with C-edge sites used for theoretical calculations of adsorption energy.

**Figure S14.** (a) Adsorption energies of Zn atom on Cu(111) surface, C-basal (top, bridge, and hollow sites), C-edge, and oxygen-containing functional groups in GONR. The C-edge site exhibited the strongest affinity energy for Zn (–2.18 eV) compared to Cu(111) (–1.20 eV), C-basal plane (–0.20 eV, –0.20 eV, and –0.22 eV for top, bridge, and hollow sites, respectively), and oxygen-containing functional groups (–0.14 eV, –0.09 eV, and –0.37 eV for epoxy, hydroxyl, and carboxyl groups, respectively). This is attributed to the localized lone-pair electrons in the C-edges that allow stronger electronic interactions with Zn atoms than the Cu(111) surface with an electron-delocalized state or the C-basal plane based on weak π-orbitals. (b) Zn adsorption configurations on GONR, representing the corresponding atomic models used for adsorption energy calculations.

**Figure S15**. Zn^2+^ adsorption as a function of MWNT oxidation time (C-edge density). The adsorbed amount of Zn^2+^ increases with oxidation time, indicating that C-edge sites play a dominant role in promoting Zn adsorption.

**Figure S16.** pDOS spectra of a Zn atom adsorbed on C-basal.

**Figure S17.** Partial density of states (pDOS) spectra of (a) two and (b) four Zn atoms adsorbed on C-edges with corresponding schematic images.

**Figure S18.** (a) Activation energy (*E*_a_) for the charge transfer reaction in Zn@Cu||Zn@Cu and Zn@OHNS-Cu||Zn@OHNS-Cu symmetric cells. Nyquist plots of (b) Zn@Cu||Zn@Cu and (c) Zn@OHNS-Cu||Zn@OHNS-Cu symmetric cells measured at temperatures ranging from 30 to 70 °C. The insets are electrical circuits of symmetric cells.

**Figure S19.** Schematic top view representation of a Zn atom(s) adsorbed on the C-edge site(s), corresponding to **Figure 3c**.

**Figure S20.** (a) Schematics of adsorption configurations (lateral vs. vertical) on Cu(111) (top) and C-edge (bottom). The inset gray box in the C-edge model represents the corresponding top view. (b) Corresponding adsorption energies of four Zn atoms to evaluate thermodynamic stability of these adsorption configurations.

**Figure S21.** Activation energy barrier for Zn diffusion calculated utilizing NEB method. The images depict intermediate steps during Zn diffusion on (a) the Cu(111) surface and (b) C-edge. The inset gray box in the C-edge model represents the corresponding top view.

**Figure S22.** CA profiles of pristine Cu and OHNS-Cu at an overpotential of −150 mV.

**Figure S23.** Voltage profiles of Zn deposition in Zn||Cu cells on (a) GO-Cu and (b) OHNS-Cu at various current densities (1, 5, 50, and 120 mA cm^−2^). (c) Comparison of nucleation overpotential between GO-Cu and OHNS-Cu.

**Figure S24.** CA profiles of GO-Cu and OHNS-Cu at an overpotential of −150 mV.

**Figure S25.** (a) CE of Zn plating/stripping in Zn||Cu cells with OHNS-Cu and GO-Cu at a current density of 120 mA cm^−2^ and an areal capacity of 2 mAh cm^−2^. (b) Corresponding Zn plating/stripping voltage profiles of Zn||Cu cells with GO-Cu.

**Figure S26.** SEM images of pristine Cu (top) and OHNS-Cu (bottom) after Zn electrodeposition at a current density of 120 mA cm^−2^ with areal capacities of 0.5 mAh cm^−2^ and 2 mAh cm^−2^.

**Figure S27.** AFM images of pristine Cu (top) and OHNS-Cu (bottom) after Zn electrodeposition at a current density of 120 mA cm^−2^ with areal capacities of 0.5 mAh cm^−2^ and 2 mAh cm^−2^.

**
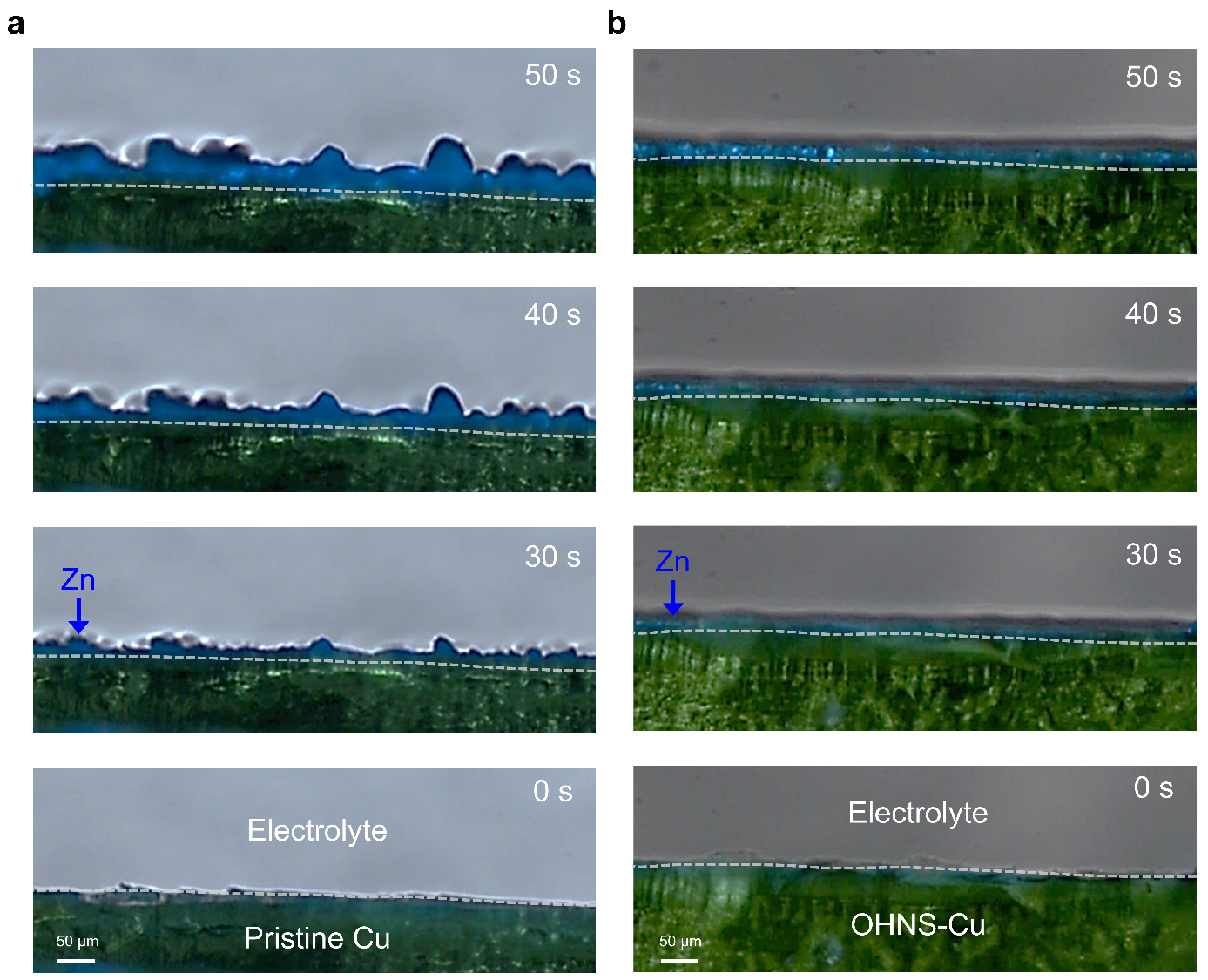
**

**Figure S28**. In situ optical microscopy (OM) images of (a) pristine Cu and (b) OHNS-Cu in Zn||Cu cells during the CA tests at −1 V (vs. Zn^2+^/Zn).

**Figure S29**. XRD patterns of (a) pristine Cu and (b) OHNS-Cu current collectors as a function of Zn deposition capacity (0.5, 1, 3, and 5 mAh cm^−2^). (c) *I*_002_/*I*_100_ and (d) *I*_002_/*I*_101_ ratios with increasing Zn deposition capacity for pristine Cu and OHNS-Cu.

**Figure S30.** Galvanostatic Zn plating/stripping profiles of Zn||Cu cells with (a) pristine Cu and (b) OHNS-Cu at a current density of 5 mA cm^−2^ and an areal capacity of 2 mAh cm^−2^.

**Figure S31.** Galvanostatic Zn plating/stripping profiles of Zn||Cu cells with (a) pristine Cu and (b) OHNS-Cu at a current density of 120 mA cm^−2^ and an areal capacity of 2 mAh cm^−2^.

**Figure S32.** (a) Electrochemical performance of Zn@Cu||Zn@Cu and Zn@OHNS-Cu||Zn@OHNS-Cu symmetric cells at a current density of 1 mA cm^−2^ with an areal capacity of 1 mAh cm^−2^. Magnified voltage hysteresis profiles of (b) Zn@Cu||Zn@Cu cell and (c) Zn@OHNS-Cu||Zn@OHNS-Cu cell.

**Figure S33.** (a) CE of Zn plating/stripping in Zn||Cu cells with pristine Cu and OHNS-Cu at a current density of 1 mA cm^−2^ and an areal capacity of 1 mAh cm^−2^. Corresponding Zn plating/stripping voltage profiles of Zn||Cu cells with (b) pristine Cu and (c) OHNS-Cu.

**Figure S34.** SEM images of Zn deposits on (a) pristine Cu and (b) OHNS-Cu in Zn||Cu cells after 50 cycles at a current density of 1 mA cm^−2^ with an areal capacity of 1 mAh cm^−2^.

**Figure S35.** SEM-EDS images of glass fiber separators obtained from Zn||Cu cells with pristine Cu (top) and OHNS-Cu (bottom) after 50 cycles at a current density of 1 mA cm^−2^ with an areal capacity of 1 mAh cm^−2^.

Dead Zn formation during cycling was evaluated by SEM and EDS analyses of glass fiber separators from cycled Zn||Cu cells shown in **Figure S33**. In pristine Cu cells, dead Zn was detected in the separator, arising from dendrite growth and break-off, whereas OHNS-Cu cells exhibited reduced dead Zn, consistent with the improved uniformity of Zn deposition.

**Figure S36.** Contact angles of water on (a) pristine Cu and (b) OHNS-Cu.

**Figure S37.** Hydrogen gas evolution profiles of pristine Cu and OHNS-Cu electrodes obtained by in situ DEMS during the cathodic scan (0.1 mV s^−1^).

**Figure S38**. Hydrogen gas evolution profiles of pristine Cu and OHNS-Cu obtained by in situ DEMS during Zn plating/stripping in Zn||Cu cells at a current density of 120 mA cm^−2^.

**Figure S39.** Contact angles of aqueous electrolyte on (a) pristine Cu and (b) OHNS-Cu.

**Figure S40**. Linear sweep voltammetry (LSV) curves of Zn@Cu and Zn@OHNS-Cu electrodes at a scan rate of 1 mV s^−1^.

**Figure S41.** XRD patterns of (a) Zn@Cu and (b) Zn@OHNS-Cu electrodes, prepared by electroplating with 2 mAh cm^−2^ of Zn onto pristine Cu and OHNS-Cu substrates at a current density of 5 mA cm^−2^, followed by aging for different durations (0, 24, and 72 h) (ZHS, JCPDS #44-0673). The cells were disassembled after the designated aging periods to examine the formation of corrosion products.

**Figure S42.** (a) Cycling performance of Zn||CVO cells. Galvanostatic charge/discharge profiles of Zn||CVO cells at a current density of 5 A g^−1^: (b) Zn@Cu and (c) Zn@OHNS-Cu.

**Figure S43.** (a) Cycling performance of Zn||CVO cells at a current density of 0.3 A g^−1^ with Zn@Cu and Zn@OHNS-Cu. Corresponding charge/discharge profiles of (b) Zn@Cu and (c) Zn@OHNS-Cu.

**Figure S44.** Galvanostatic charge/discharge profiles of anode-free full cells with (a) pristine Cu and (b) OHNS-Cu at various current densities (*j*) (0.1, 1, 2, 5, and 10 A g^−1^).

**Figure S45.** (a) Cycling performance of anode-free full cells at a current density of 1 A g^−1^. Corresponding charge/discharge profiles of anode-free full cells: (b) pristine Cu and (c) OHNS-Cu.

**Figure S46.** Cycle number versus capacity retention for anode-free Zn pouch cells: OHNS-Cu (this work) and previously reported anode-free Zn cells.

**Figure S47.** SEM images of (a) pristine Cu after 84 cycles and (b) OHNS-Cu after 150 cycles in anode-free full cells, both cycled at a current density of 10 A g^−1^.


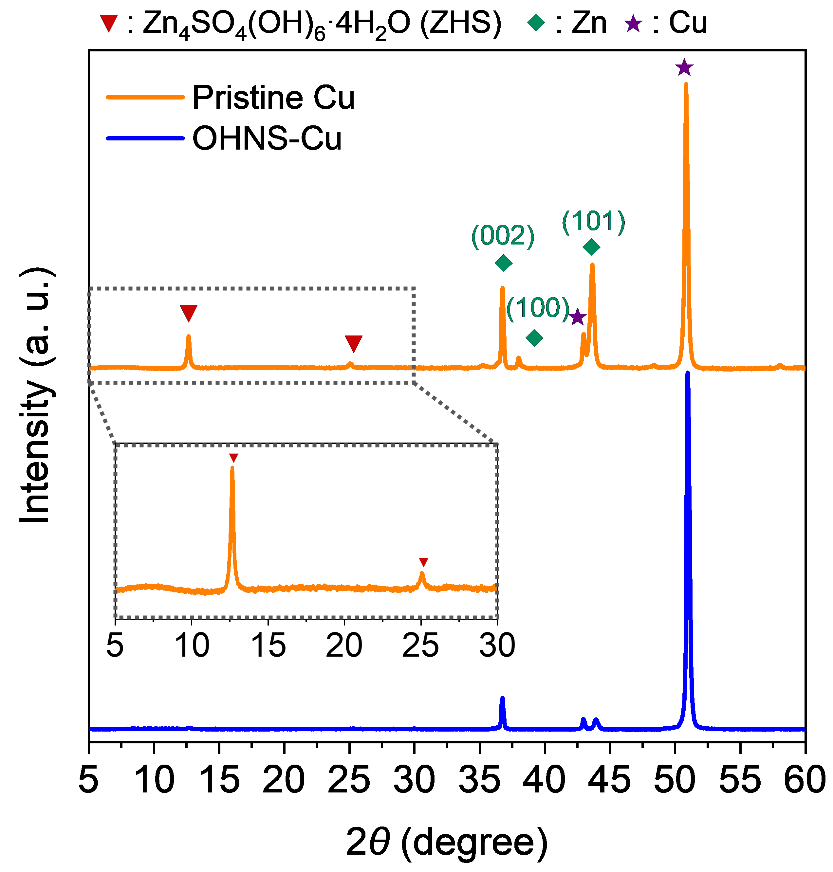


**Figure S48**. XRD patterns of pristine Cu and OHNS-Cu after 50 cycles in the anode-free full cell at a current density of 10 A g^−1^. The inset shows an enlarged view of the XRD pattern of pristine Cu.


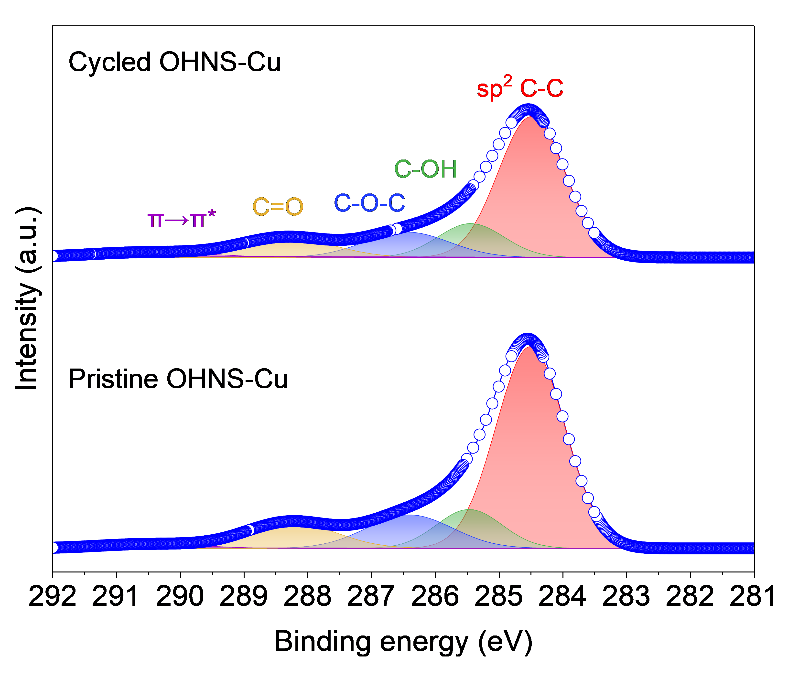


**Figure S49**. C 1*s* XPS spectra of pristine OHNS-Cu and cycled OHNS-Cu obtained after 50 cycles in the anode-free full cell at 10 A g ^−1^.

**Table S1.** Comparison of substrate design strategies between this work and previously reported studies.

| **Ref.** | **Coating**  **method** | **Coating**  **thickness** | **Cell Configuration**  **(Cell-type)** | **NP**  **ratio** | **Capacity retention/cycle number** | **Supporting Information Ref.** |
| --- | --- | --- | --- | --- | --- | --- |
| This  work | Slot-die  coating | 80 nm | Zn@OHNS-Cu\|\|CVO  (Coin) | 7.7 | 91.8%/3000^th^  (at 5 A g^−1^) | This  work |
|  |  |  | OHNS-Cu\|\|Zn*_x_*CVO  (Pouch) | Anode-free | 82.2% / 800th  (at 10 A g^−1^) |  |
| [37] | Drop  casting | 25 μm | PZS-Zn\|\|NVO  (Pouch) | 9.97 | 71.94%/200^th^  (at 1 A g^−1^) | [3] |
| [38] |  | 10 μm | PSN-Zn\|\|BVO  (Coin) | N/A | 94.5%/800^th^  (at 4 A g^−1^) | [4] |
| [39] |  | 4 μm | ZrO_2_-Zn\|\|V_2_O_5_  (Coin) | N/A | 42.3%/3000^th^  (at 1 A g^−1^) | [5] |
| [40] | Spray  coating | 1.1 μm | MPVMT@Zn\|\|I_2_  (Coin) | 1.9 | ~100% / 200^th^  (at 5 mA cm^−2^) | [6] |
| [41] |  | N/A | Zn@Act-LDH-H@SS\|\|MnO_2_ (Coin) | N/A | >100% / 1800^th^  (at 2 A g^−1^) | [7] |
|  |  |  | Zn@Act-LDH-H@SS\|\|MnO_2_ (Pouch) | N/A | ~84.2% / 80^th^  (at 0.24 A g^−1^) |  |
| [42] |  | N/A | VZSe/V@Zn\|\|NVO (Pouch) | 3.5 | 90.3% / 400^th^  (at 1 A g^−1^) | [8] |
| [43] | Doctor  blading | 10 μm | LAP-Zn\|\|modified-MnO_2_ (Coin) | N/A | ~71.4% / 200^th^  (at 2 A g^−1^) | [9] |
| [44] |  | N/A | Zn@GSS\|\|α-MnO_2_  (Coin) | 2 | ~55.6%/1000^th^  (at 8 mA cm^−2^) | [10] |
| [45] |  | 20 μm | Zn@ZVO\|\|V_2_O_5_  (Coin) | N/A | 79.1%/1000^th^  (at 2 A g^−1^) | [11] |
| [47] | Chemical vapor  deposition | 4 μm | ZnTe@Zn\|\|MnO_2_  (Coin) | 3 | 34.3%/500^th^  (at 0.616 A g^−1^) | [12] |
| [48] | Solvothermal-pulling  method | 100 nm | FCOF@Zn\|\|MnO_2_  (Coin) | 5 | ~59.3%/300^th^  (at 4 mA cm^−2^) | [13] |

Abbreviations

- PZS: Ion-conductive layer based on polyvinyl alcohol substrate via the synergistic embedded of an

anion soluble zinc salt Zn(CF_3_SO_3_)_2_ and Si_3_N_4_ nanoparticles

- PSN: Polyacrylonitrile
- BVO: Ba_0.26_V_2_O_5_·0.92H_2_O
- MPVMT: Monolayer porous vermiculites
- Act-LDH-H: Horizontal activated layered double hydroxide
- SS: Stainless steel
- VZSe/V: Dual-layer structure consisting of the VSe_2_-ZnSe outer layer and nanometallic V inner layer
- NVO: Ammonium vanadate (NH_4_V_4_O_10_)
- LAP: Laponite film
- GSS: Graphene coated stainless steel
- ZVO: Zinc pyrovanadate
- ZnTe: Zinc telluride
- FCOF: Fluorinated 2D porous covalent organic framework

**Table S2.** Comparison of cell performance between this study and previously reported anode-free full cells.

| **Ref.** | **Strategy** | **Cell configuration**  **(Cell type)** | **Electrolyte** | **Specific capacity [mAh g^−1^] at charge/discharge current density [A g^−1^]** | **Capacity retention / cycle number** | **Supporting Information Ref.** |
| --- | --- | --- | --- | --- | --- | --- |
| This work | Substrate  design | OHNS-Cu\|\|Zn*_x_*CVO  (Coin) | 3.4 m ZnSO_4_ in H_2_O | 251.3 (at 0.1 A g^−1^)  216.2 (at 1 A g^−1^)  186.5 (at 2 A g^−1^)  132.6 (at 5 A g^−1^)  94.2 (at 10 A g^−1^) | 71.3% / 100^th^  (at 1 A g^−1^)  86.3% / 150^th^  (at 10 A g^−1^) | This work |
|  |  | OHNS-Cu\|\|Zn*_x_*CVO  (Pouch) |  | 95.1 (at 10 A g^−1^) | 82.2% / 800^th^  (at 10 A g^−1^) |  |
| [42] |  | VZSe/V@Cu\|\|ZnV_3_O_8_  (Pouch) | 2 M ZnSO_4_ in H_2_O | ~67.3 (at 2 A g^−1^) | ~76% / 300^th^  (at 2 A g^−1^) | [8] |
| [31] |  | Cu@Cu_3_Zn-CCF\|\|Zn_3_V_3_O_8_  (Coin) | 3 M Zn(OTf)_2_ in H_2_O | 114 (at 0.1 A g^−1^)  58 (at 2 A g^−1^) | 80% / 200^th^  (at 2 A g^−1^) | [14] |
| [117] |  | Cu@Na-MX@Sn\|\|LiMn_2_O_4_  (Coin) | 2 M ZnSO_4_ + 1 M Li_2_SO_4_ in H_2_O | ~ 60 (at 0.1 A g^−1^)  ~37 (at 0.2 A g^−1^) | 74.0% / 100^th^  (at 0.1 A g^−1^)  68.9% / 100^th^  (at 0.2 A g^−1^) | [15] |
| [118] |  | Cu@PMMA:Zn\|\|ZnMnO_2_  (Coin) | 3 M Zn(OTf)_2_ in H_2_O | 257 (at 1 A g^−1^) | 80.0% / 300^th^  (at 1 A g^−1^) | [16] |
| [64] |  | CuNC@Cu\|\| G/PVP@ZnI_2_  (Coin) | 2 M ZnSO_4_ + 0.005 M ZnI_2_ + 0.01 M I_2_ in H_2_O | 125.7 (at 1 A g^−1^) | 63.8% / 200^th^  (at 1 A g^−1^) | [17] |
| [19] | Electrolyte additive | SS\|\|LiMn_2_O_4_  (Coin) | 2 M ZnSO_4_ + 0.08 M ZnF_2_ in H_2_O | 91.4 (at 0.1 A g^−1^)  77.3 (at 0.2 A g^−1^)  63.2 (at 0.5 A g^−1^)  54.5 (at 0.8 A g^−1^)  49.5 (at 1.0 A g^−1^) | 75.6% / 100^th^  (at 0.2 A g^−1^)  76.8% / 100^th^  (at 0.4 A g^−1^) | [18] |
| [26] |  | Ti\|\|Zn_x_VOPO_4_  (Pouch) | 4 m Zn(OTf)_2_ + 0.5 m Me_3_EtNOTF in H_2_O | ~ 135 (at 0.5 mA cm^−2^) | > 80% / 90^th^  (at 0.5 mA cm^−2^) | [19] |
| [27] |  | Cu\|\|LFP  (Coin) | 4 M ZnSO_4_ + 2 M Li_2_SO_4_ + 0.005 M SnBr_2_ in H_2_O/DME (volume ratio, 9/1) | 126 (at 0.085 A g^−1^)  N/A (at 0.17 A g^−1^)  N/A (at 0.34 A g^−1^) | 35.2% / 100^th^  (at ~ 0.085 A g^−1^)  14.8% / 100^th^  (at ~ 0.17 A g^−1^)  12.8% / 100^th^  (at ~ 0.34 A g^−1^) | [20] |
| [119] |  | Cu\|\|Zn_x_PANI  (Coin) | 1 M ZnSO_4_ + 4M EMImCl in H_2_O | 154.4 (at 1.0 A g^−1^) | 78.8% / 300^th^  (at 1.0 A g^−1^) | [21] |
| [120] |  | Cu\|\|Zn_x_MnVO  (Coin) | 2 M ZnSO_4_ + 0.04 M FU in H_2_O | ~200 (at 2.0 A g^−1^) | ~35% / 270^th^  (at 2.0 A g^−1^) | [22] |
| [28] | Electrolyte cosolvent | Cu\|\|ZnMn_2_O_4_  (Coin) | 1 M Zn(OTf)_2_ in H_2_O/PC (volume ratio, 1/1) | ~ 90 (at 0.35 A g^−1^) | 80% / 275^th^  (at 0.35 A g^−1^) | [23] |
| [121] |  | Cu\|\|LFP/C  (Coin) | 2 M Zn(OTf)_2_ + 1 M LiOTf in H_2_O/EG (volume ratio, 3/7) | ~ 120 (at 0.33 A g^−1^) | 75.2% / 100^th^  (at 0.33 A g^−1^) | [24] |
| [29] | Hybrid eutectic electrolyte | Cu\|\|Zn_0.25+x_VO  (Swagelok) | 2 m Zn(OTf)_2_ in Sulfolane/H_2_O (weight ratio, 7/3) | 113.5 (at 0.045 A g^−1^) | 85% / 100^th^  (at 0.045 A g^−1^) | [25] |
| [62] | Biphasic electrolyte | gr-Cu\|\|Zn_x_CVO  (Coin) | 3.4 m ZnSO_4_ in H_2_O  0.5 m Zn(TFSI)_2_ in AN | 251.2 (at 0.1 A g^−1^)  197.8 (at 0.3 A g^−1^)  176.2 (at 0.5 A g^−1^)  140.9 (at 1.0 A g^−1^) | 76.7% / 100^th^  (at 1 A g^−1^) | [26] |
| [112] | Separator modification | Cu\|\|NaV_3_O_8_·  1.5H_2_O  (Coin) | 2 M ZnSO_4_ in H_2_O  w/ HTO@GF separator | ~ 200 (at 0.2 A g^−1^) | 62.5% / 150^th^  (at 0.2 A g^−1^) | [27] |

Abbreviations

- Zn(OTf)_2_: Zinc trifluoromethanesulfonate (Zn(CF_3_SO_3_)_2_)
- Cu@Cu_3_Zn-CCF: Carbon-coated Cu
- Na-MX@Sn: Na^+^ decorated MXene nanosheets with in situ anchoring ultrafine Sn nanodots
- PMMA:Zn: Polymethylmethacrylate matrix containing Zn ions
- CuNC: Cu nanoclusters
- G/PVP: Graphene/polyvinyl pyrrolidone
- Ti: Titanium
- LFP: Lithium iron phosphate (LiFePO_4_)
- DME: 1,2-dimethoxyethane
- PANI: Polyaniline
- EMImCl: 1-ethyl-3-methylimidazolium chloride
- MnVO: Manganese vanadate
- FU: Fumaric acid
- PC: Propylene carbonates
- EG: Ethylene glycol
- gr-Cu: Cu foil modified with a graphene monolayer
- Zn(TFSI)_2_: Zinc(II) bis(trifluoromethanesulfonyl)imide (Zn(C_2_F_6_NO_4_S_2_)_2_)
- AN: Acetonitrile
- HTO@GF: glass fiber separator with hydrated titanic acid

**Table S3.** Comparison of cell performance between this study and previously reported Zn-based full cells.

| **Ref.** | **Strategy** | **Cathode** | **Anode** | **Electrolyte** | **Specific capacity [mAh g^−1^] at charge/discharge current density [A g^−1^]** | **N/P ratio** | **Supporting Information Ref.** |
| --- | --- | --- | --- | --- | --- | --- | --- |
| This work | Substrate design | Zn*_x_*CVO | OHNS-Cu | 3.4 m ZnSO_4_ in H_2_O | 251.3 (at 0.1 A g^−1^)  216.2 (at 1 A g^−1^)  186.5 (at 2 A g^−1^)  132.6 (at 5 A g^−1^)  94.2 (at 10 A g^−1^) | Anode-free | This work |
|  |  | CVO | Zn@OHNS-Cu |  | 125.7 (at 5 A g^−1^) | 7.7 |  |
| [53] |  | MnO_2_ | Zn(002)@ZPO | 2 M ZnSO_4_ in H_2_O | 188 (at 0.92 A g^−1^) | 62.3 | [28] |
| [54] |  | MnO_2_ | ZnCu@Zn | 2 M ZnSO_4_ in H_2_O | 192 (at 0.5A g^−1^) | 50.8 | [29] |
| [55] |  | NVPF@C | Zn@C | 8 M NaClO_4_ + 0.4 M Zn(OTf)_2_ | 63.1 (at 0.1 A g^−1^) | 156 | [30] |
| [60] |  | NH_4_V_4_O_10_ | Zn-PG | 3 M Zn(OTf)_2_ in H_2_O | 410 (at 0.1 A g^−1^) | 5.7 | [31] |
| [52] | Electrolyte modification | V_2_O_5_ | Zn | 2 M ZnSO_4_ in H_2_O + β-CD | 307.5 (at 0.5 A g^−1^) | 47.6 | [32] |
| [56] |  | PQ-MCT | Zn | 1 m Zn(OTf)_2_ in DMF | 145 (at 0.05 A g^−1^) | 15 | [33] |
| [57] |  | V_2_O_5_ | Zn | 4 m Zn(BF_4_)_2_/EG | 145 (at 1 A g^−1^) | 22.9 | [34] |
| [59] |  | VS_2_@SS | Zn | 2 M Zn(OTf)_2_ + 25 mM ZnI_2_ in H_2_O/DME (volume ratio, 5/5) | ~195 (at 0.1 A g^−1^) | 3.6 | [35] |
| [61] |  | VOPO_4_⋅2H_2_O | Zn | 30 m ZnCl_2_ + 5 m LiCl + 10 m TMACl in H_2_O/DMC (molar ratio, 5/1) | ~120 (at 0.05 A g^−1^) | 2.3 | [36] |
| [63] |  | V_2_O_5_ | Zn | 1 m Zn (OTf)_2_ + 0.025 m Zn(H_2_PO_4_)_2_  in H_2_O | 104.2 (at 0.8 A g^−1^) | 2.3 | [37] |
| [58] | Intercalation anode | Zn_0.2_MnO_2_ | h-MoO_3_ | 1 M ZnSO_4_ in H_2_O | 56.7 (at 0.1 A g^−1^) | 0.9 | [38] |

Abbreviations

- AC: Activated carbon
- ZPO: Zn_3_(PO_4_)_2_
- ZnCu@Zn: Heterostructured interface composed of ZnO rod array and CuZn_5_ layer on the Zn
- Zn@C: Active carbon coated Zn
- NVPF@C: Carbon-coated Na_3_V_2_(PO_4_)_2_F_3_
- Zn-PG: Polymer glue-coated Zn
- β-CD: β-cyclodextrin
- PQ-MCT: Phenanthrenequinone macrocyclic trimer
- DMF: N,N-dimethylformamide
- EG: Ethylene glycol
- TMACl: Trimethylammonium chloride
- DMC: Dimethyl carbonate
- h-MoO_3_: Hexagonal MoO_3_

**Table S4.** Calculation details for the energy/power densities of anode-free Zn cells containing the OHNS-Cu electrode, determined based on the total mass of both electrodes. The mass loading of cathode includes active material, conductive material, and binder material.

| **Cathode electrode** | **Anode electrode** | **Electrolyte** | **Mass loading of cathode**  **[mg cm^−2^]** | **Mass loading of anode**  **[mg cm^−2^]** | **Areal capacity**  **[mAh cm^−2^]** | **Operating voltage [V]** | **Current density**  **[A g^−1^]** | **Discharge time [h]** | **Energy density**  **[Wh kg^−1^]** | **Power density**  **[W kg^−1^]** |
| --- | --- | --- | --- | --- | --- | --- | --- | --- | --- | --- |
| Zn*_x_*CVO | OHNS-Cu | 3.4 m ZnSO_4_ in H_2_O | 3.51 | 0.91 | 0.62 | 1.00 | 0.1 | 2.53 | 140.63 | 55.61 |
|  |  |  | 3.55 | 1.03 | 0.54 | 0.91 | 1 | 0.22 | 108.29 | 493.40 |
|  |  |  | 2.29 | 0.67 | 0.30 | 0.90 | 2 | 0.09 | 90.78 | 973.42 |
|  |  |  | 3.44 | 0.80 | 0.31 | 0.85 | 5 | 0.03 | 63.16 | 2414.70 |
|  |  |  | 3.31 | 0.89 | 0.22 | 0.75 | 10 | 0.01 | 38.85 | 4138.09 |

Energy density of the Zn full cell was calculated based on the **Equation S2** [39].

$$Energy density [Wh \mathrm{kg}^{-1}]=\frac{Discharge energy}{Mass of electrodes}=\frac{Nominal Voltage \times C/A}{M_{\mathrm{cathode}}/A+M_{\mathrm{anode}}/A} (2)$$

*M*_cathode_​ (mg) refers to the mass of the cathode, including the active material, conductive additive, and binder. *M*_anode_​ (mg) denotes the Zn mass, which was calculated based on the Zn source present in the pre-zincificated cathode for Zn anode-free full cells, and the mass of the Zn metal anode for conventional Zn metal full cells. *C* (mAh) and *A* (cm^2^) represent the capacity and electrode area, respectively.

The power density of the Zn full cell was calculated based on the **Equation S3** [40].

$$Power density \left( W \mathrm{kg}^{-1} \right)= \frac{Energy density \left( \mathrm{Wh}\mathrm{kg}^{-1} \right)}{Discharge time (h)} \left( 3 \right)$$

**Table S5.** Possible adsorption sites and corresponding adsorption energies of Zn on Cu(111) surface.

| **Site** | **Hollow, fcc** | **Hollow, hcp** | **Atop** | **Bridge** |
| --- | --- | --- | --- | --- |
| *E*_ads_ | -1.199 | -1.193 | -1.089 | -1.085 |

**Table S6.** Comparison between this study and previously reported Zn||Cu asymmetric cells.

| **Ref.** | **Strategy** | **Cell configurations** | **Electrolyte** | **Current density (mA cm**^−2^**)**  **/Areal capacity (mAh cm**^−2^**)** | **CE/Cycle number** | **Supporting Information Ref.** |
| --- | --- | --- | --- | --- | --- | --- |
| This work | Substrate  design | OHNS-Cu\|\|Zn | 2 M ZnSO_4_ in H_2_O | 120/2 | 99.5%/250 | This work |
| [37] |  | Cu\|\|PZS-Zn | 2 M ZnSO_4_ in H_2_O | 0.25/0.25 | 99.8%/1400 | [3] |
| [43] |  | LAP-Ti\|\|Zn | 2 M ZnSO_4_ in H_2_O | 0.5/0.5 | 99.1%/500 | [9] |
| [40] |  | MPVMT@Ti\|\|Zn | 2 M ZnSO_4_ in H_2_O | 2/1 | 99.4%/400 | [6] |
| [41] |  | Act-LDH-H@SS\|\|Zn | 2 M ZnSO_4_ in H_2_O | 5/0.5 | 99.55%/8000^th^ | [7] |
| [44] |  | GSS\|\|Zn | 2 M ZnSO_4_ in H_2_O | 4/0.8 | >99%/10000^th^ | [10] |
| [45] |  | ZVO@Ti\|\|Zn | 2 M ZnSO_4_ in H_2_O | 2/1 | 98.2%/200^th^ | [11] |
| [38] |  | PSN-Ti\|\|Zn | 2 M ZnSO_4_ in H_2_O | 5/1 | 98.7%/200^th^ | [4] |
| [39] |  | Ti\|\|ZrO_2_-Zn | 2 M ZnSO_4_ in H_2_O | 20/5 | 99.36%/230^th^ | [5] |
| [48] |  | FCOF-Ti\|\|Zn | 2 M ZnSO_4_ in H_2_O | 40/2  80/1 | 97.3%/250  97.2%/320 | [13] |
| [97] | Electrolyte  modification | Cu\|\|Zn | 2 M ZnSO_4_ + Irgacure 2959 in H_2_O | 1/0.5 | 99.8%/3000 | [41] |
| [102] |  | Cu\|\|Zn | 1 M ZnSO_4_ + 0.01 M TA-Na in H_2_O | 0.2/0.1 | 99.3%/1000 | [42] |
| [103] |  | Cu\|\|Zn | 1 M ZnSO_4_ + 0.01 M SPS in H_2_O | 1/1 | 99.72%/1200 | [43] |
| [104] |  | Ti\|\|Zn | 1.3 m ZnCl_2_ in H_2_O/DMSO (volume ratio, 4.3/1) | 1/0.5 | 99.5%/400^th^ | [44] |
| [105] |  | Cu\|\|Zn | 2 M ZnSO_4_ + 0.02 M SeO_2_ in H_2_O | 2/2 | 99.5%/250^th^ | [45] |
| [106] |  | Cu\|\|Zn | 0.75 M Zn(OTf)_2_ in H_2_O/DMI | 3/1.5 | 98.16%/500^th^ | [46] |

**Table S7.** Bader charge and estimated electrostatic interaction.

| **System** | **Perpendicular  distance (*r*)** | **H in H_2_O (*Q*_1_)^a^** | **C or Cu (*Q*_2_)^b^** | **Estimated  electrostatic energy**  **(*Q*_1_*Q*_2_ *r*^−1^ 10^−3^)** |
| --- | --- | --- | --- | --- |
| Cu(111) | 2.63377Å | 0.5417e | -0.00745e | -1.532 |
| C-edge | 2.65138Å | 0.5564e | 0.00397e | 0.833 |

^a,b^: The *Q*_1_ and *Q*_2_ values were determined by averaging the Bader charges of each atom. For C and Cu, only atoms on the surface in each structure were considered in the calculations.

**References**

1. Kim, J. H., Kim, W. Y., Kim, S., Kim, J., Lee, S. J., Park, N., Han, S. P., Ryu, K., Kim, J., Lee, W. B. & Lee, S. Y. Kosmotropic aqueous processing solution for green lithium battery cathode manufacturing. *Nat. Commun.* **16**, 1686 (2025).

2. Heaney, M. B. Electrical Conductivity and Resistivity. in *Electrical Measurement, Signal Processing, and Displays* (ed. John G. Webster) (CRC Press, Boca Raton, 2004).

3. Meng, X., Zhou, S., Li, J., Chen, Y., Lin, S., Han, C. & Pan, A. Regulated Ion-Conductive Electrode–Electrolyte Interface by In Situ Gelation for Stable Zinc Metal Anode. *Adv. Funct. Mater.* **34**, 2309350 (2023).

4. Zhou, S., Wang, Y., Lu, H., Zhang, Y., Fu, C., Usman, I., Liu, Z., Feng, M., Fang, G., Cao, X., Liang, S. & Pan, A. Anti-Corrosive and Zn-Ion-Regulating Composite Interlayer Enabling Long-Life Zn Metal Anodes. *Adv. Funct. Mater.* **31**, 2104361 (2021).

5. Liang, P., Yi, J., Liu, X., Wu, K., Wang, Z., Cui, J., Liu, Y., Wang, Y., Xia, Y. & Zhang, J. Highly Reversible Zn Anode Enabled by Controllable Formation of Nucleation Sites for Zn-Based Batteries. *Adv. Funct. Mater.* **30**, 1908528 (2020).

6. Zheng, Z., Zhong, X., Zhang, Q., Zhang, M., Dai, L., Xiao, X., Xu, J., Jiao, M., Wang, B., Li, H., Jia, Y., Mao, R. & Zhou, G. An extended substrate screening strategy enabling a low lattice mismatch for highly reversible zinc anodes. *Nat. Commun.* **15**, 753 (2024).

7. Zhang, S., Li, J., Jin, B. & Shao, M. Oriented Zinc Metal Anode Based on Directional Recognition and Assembly. *Small* **19**, 2301874 (2023).

8. Zhang, H., You, Y., Sha, D., Shui, T., Moloto, N., Liu, J., Kure-Chu, S. Z., Hihara, T., Zhang, W. & Sun, Z. M. Planar Deposition via In Situ Conversion Engineering for Dendrite-Free Zinc Batteries. *Adv. Mater.* **36**, 2409763 (2024).

9. Feng, J., Li, X., Cui, X., Zhao, H., Xi, K. & Ding, S. Periodically Alternating Electric Field Layers Induces the Preferential Growth of Zn (002) Plane for Ultralow Overpotential Zinc-Ion Batteries. *Adv. Energy Mater.* **13**, 2204092 (2023).

10. Zheng, J., Zhao, Q., Tang, T., Yin, J., Quilty, C. D., Renderos, G. D., Liu, X., Deng, Y., Wang, L., Bock, D. C., Jaye, C., Zhang, D., Takeuchi, E. S., Takeuchi, K. J., Marschilok, A. C. & Archer, L. A. Reversible epitaxial electrodeposition of metals in battery anodes. *Science* **366**, 645–648 (2019).

11. Zhou, Y., Li, G., Feng, S., Qin, H., Wang, Q., Shen, F., Liu, P., Huang, Y. & He, H. Regulating Zn Ion Desolvation and Deposition Chemistry Toward Durable and Fast Rechargeable Zn Metal Batteries. *Adv. Sci.* **10**, 2205874 (2023).

12. Wang, R., Xin, S., Chao, D., Liu, Z., Wan, J., Xiong, P., Luo, Q., Hua, K., Hao, J. & Zhang, C. Fast and Regulated Zinc Deposition in a Semiconductor Substrate toward High-Performance Aqueous Rechargeable Batteries. *Adv. Funct. Mater.* **32**, 2207751 (2022).

13. Zhao, Z., Wang, R., Peng, C., Chen, W., Wu, T., Hu, B., Weng, W., Yao, Y., Zeng, J., Chen, Z., Liu, P., Liu, Y., Li, G., Guo, J., Lu, H. & Guo, Z. Horizontally arranged zinc platelet electrodeposits modulated by fluorinated covalent organic framework film for high-rate and durable aqueous zinc ion batteries. *Nat. Commun.* **12**, 6606 (2021).

14. Xie, S., Li, Y. & Dong, L. Stable anode-free zinc-ion batteries enabled by alloy network-modulated zinc deposition interface. *J. Energy Chem.* **76**, 32–40 (2023).

15. An, Y., Xu, B., Tian, Y., Shen, H., Man, Q., Liu, X., Yang, Y. & Li, M. Reversible Zn electrodeposition enabled by interfacial chemistry manipulation for high-energy anode-free Zn batteries. *Mater. Today* **70**, 93–103 (2023).

16. Jeong, D. Y., Chang, W. J., Jang, S., Kim, M., Kim, Y., Kim, B. & Park, W. Il. Controlling dendrite growth and side reactions in anode-free Zn-ion aqueous batteries with PMMA:Zn coated electrode. *J. Energy Storage* **76**, 109791 (2024).

17. Zhang, Y., Wang, L., Li, Q., Hu, B., Kang, J., Meng, Y., Zhao, Z. & Lu, H. Iodine Promoted Ultralow Zn Nucleation Overpotential and Zn-Rich Cathode for Low-Cost, Fast-Production and High-Energy Density Anode-Free Zn-Iodine Batteries. *Nano-Micro Lett.* **14**, 208 (2022).

18. An, Y., Tian, Y., Zhang, K., Liu, Y., Liu, C., Xiong, S., Feng, J. & Qian, Y. Stable Aqueous Anode-Free Zinc Batteries Enabled by Interfacial Engineering. *Adv. Funct. Mater.* **31**, 2101886 (2021).

19. Cao, L., Li, D., Pollard, T., Deng, T., Zhang, B., Yang, C., Chen, L., Vatamanu, J., Hu, E., Hourwitz, M. J., Ma, L., Ding, M., Li, Q., Hou, S., Gaskell, K., Fourkas, J. T., Yang, X. Q., Xu, K., Borodin, O. & Wang, C. Fluorinated interphase enables reversible aqueous zinc battery chemistries. *Nat. Nanotechnol.* **16**, 902–910 (2021).

20. Nigatu, T. A., Bezabh, H. K., Jiang, S. K., Taklu, B. W., Nikodimos, Y., Yang, S. C., Wu, S. H., Su, W. N., Yang, C. C. & Hwang, B. J. An anode-free aqueous hybrid batteries enabled by in-situ Cu/Sn/Zn alloy formation on pure Cu substrate. *Electrochim. Acta* **443**, 141883 (2023).

21. Zhang, Q., Ma, Y., Lu, Y., Zhou, X., Lin, L., Li, L., Yan, Z., Zhao, Q., Zhang, K. & Chen, J. Designing Anion-Type Water-Free Zn^2+^ Solvation Structure for Robust Zn Metal Anode. *Angew. Chem. Int. Ed.* **60**, 23357–23364 (2021).

22. Huang, S., Fu, H., Kwon, H. M., Kim, M. S., Zhang, J. D., Lu, J., Kim, J. S., Jang, G., Min, D. H., Il Kim, W., Wang, G., Li, W., Zhang, R., Jo, S. B., Chen, X., Zhang, Q., Xu, K., Armand, M. & Park, H. S. Stereoisomerism of multi-functional electrolyte additives for initially anodeless aqueous zinc metal batteries. *Nat. Commun.* **16**, 6117 (2025).

23. Ming, F., Zhu, Y., Huang, G., Emwas, A. H., Liang, H., Cui, Y. & Alshareef, H. N. Co-Solvent Electrolyte Engineering for Stable Anode-Free Zinc Metal Batteries. *J. Am. Chem. Soc.* **144**, 7160–7170 (2022).

24. Duan, J., Min, L., Wu, M., Yang, T., Chen, M. & Wang, C. “Anode-free” Zn/LiFePO_4_ aqueous batteries boosted by hybrid electrolyte. *J. Ind. Eng. Chem.* **114**, 317–322 (2022).

25. Li, C., Kingsbury, R., Thind, A. S., Shyamsunder, A., Fister, T. T., Klie, R. F., Persson, K. A. & Nazar, L. F. Enabling selective zinc-ion intercalation by a eutectic electrolyte for practical anodeless zinc batteries. *Nat. Commun.* **14**, 3067 (2023).

26. Kim, W. Y., Kim, H. I., Lee, K. M., Shin, E., Liu, X., Moon, H., Adenusi, H., Passerini, S., Kwak, S. K. & Lee, S. Y. Demixing the miscible liquids: toward biphasic battery electrolytes based on the kosmotropic effect. *Energy Environ. Sci.* **15**, 5217–5228 (2022).

27. Zheng, Q., Lu, H., Hu, Z., Liu, L., Tang, Z., Han, C. & Li, W. Multifunctional Separator Design for Regulating Zn^2+^ Ion Flux and Zn Stripping in Anode-Less Zn Metal Batteries. *Nano Lett.* **25**, 11961–11969 (2025).

28. Song, X., Bai, L., Wang, C., Wang, D., Xu, K., Dong, J., Li, Y., Shen, Q. & Yang, J. Synergistic Cooperation of Zn(002) Texture and Amorphous Zinc Phosphate for Dendrite-Free Zn Anodes. *ACS Nano* **17**, 15113–15124 (2023).

29. Jiang, Z., Yin, K., Pan, R., Zhang, G., Cui, F., Luo, K., Xiong, Y. & Sun, L. Heterostructured Interface Enables Uniform Zinc Deposition for High-Performance Zinc-Ion Batteries. *Small* **19**, 2302995 (2023).

30. Li, W., Wang, K., Zhou, M., Zhan, H., Cheng, S. & Jiang, K. Advanced Low-Cost, High-Voltage, Long-Life Aqueous Hybrid Sodium/Zinc Batteries Enabled by a Dendrite-Free Zinc Anode and Concentrated Electrolyte. *ACS Appl. Mater. Interfaces* **10**, 22059–22066 (2018).

31. Jiao, Y., Li, F., Jin, X., Lei, Q., Li, L., Wang, L., Ye, T., He, E., Wang, J., Chen, H., Lu, J., Gao, R., Li, Q., Jiang, C., Li, J., He, G., Liao, M., Zhang, H., Parkin, I. P., Peng, H. & Zhang, Y. Engineering Polymer Glue towards 90% Zinc Utilization for 1000 Hours to Make High-Performance Zn-Ion Batteries. *Adv. Funct. Mater.* **31**, 2107652 (2021).

32. Meng, C., He, W., Jiang, L., Huang, Y., Zhang, J., Liu, H. & Wang, J. J. Ultra-Stable Aqueous Zinc Batteries Enabled by β-Cyclodextrin: Preferred Zinc Deposition and Suppressed Parasitic Reactions. *Adv. Funct. Mater.* **32**, 2207732 (2022).

33. Wang, N., Dong, X., Wang, B., Guo, Z., Wang, Z., Wang, R., Qiu, X. & Wang, Y. Zinc–Organic Battery with a Wide Operation-Temperature Window from −70 to 150 °C. *Angew. Chem. Int. Ed.* **59**, 14577–14583 (2020).

34. Han, D., Cui, C., Zhang, K., Wang, Z., Gao, J., Guo, Y., Zhang, Z., Wu, S., Yin, L., Weng, Z., Kang, F. & Yang, Q. H. A non-flammable hydrous organic electrolyte for sustainable zinc batteries. *Nat. Sustain.* **5**, 205–213 (2022).

35. Zhou, K., Li, Z., Qiu, X., Yu, Z. & Wang, Y. Boosting Zn Anode Utilization by Trace Iodine Ions in Organic-Water Hybrid Electrolytes through Formation of Anion-rich Adsorbing Layers. *Angew. Chem. Int. Ed.* **62**, e202309594 (2023).

36. Jiang, H., Tang, L., Fu, Y., Wang, S., Sandstrom, S. K., Scida, A. M., Li, G., Hoang, D., Hong, J. J., Chiu, N. C., Stylianou, K. C., Stickle, W. F., Wang, D., Li, J., Greaney, P. A., Fang, C. & Ji, X. Chloride electrolyte enabled practical zinc metal battery with a near-unity Coulombic efficiency. *Nat. Sustain.* **6**, 806–815 (2023).

37. Zeng, X., Mao, J., Hao, J., Liu, J., Liu, S., Wang, Z., Wang, Y., Zhang, S., Zheng, T., Liu, J., Rao, P. & Guo, Z. Electrolyte Design for In Situ Construction of Highly Zn^2+^-Conductive Solid Electrolyte Interphase to Enable High-Performance Aqueous Zn-Ion Batteries under Practical Conditions. *Adv. Mater.* **33**, 2007416 (2021).

38. Xiong, T., Zhang, Y., Wang, Y., Lee, W. S. V. & Xue, J. Hexagonal MoO_3_ as a zinc intercalation anode towards zinc metal-free zinc-ion batteries. *J. Mater. Chem.* **8**, 9006–9012 (2020).

39. Zhang, B., Yao, J., Wu, C., Li, Y., Liu, J., Wang, J., Xiao, T., Zhang, T., Cai, D., Wu, J., Seh, Z. W., Xi, S., Wang, H., Sun, W., Wan, H. & Fan, H. J. Electrolyte design for reversible zinc metal chemistry. *Nat. Commun.* **16**, 71 (2025).

40. Chen, X., Su, H., Liu, X., Su, L., Lan, L., Yang, B. & Liu, Q. Introduction of Electron-Withdrawing Groups in Organic Cathodes for Enhancing the Performance of Air-Charging Aqueous Zinc-Ion Batteries. *Energy Fuels* **39**, 14910–14920 (2025).

41. Li, T. C., Lin, C., Luo, M., Wang, P., Li, D. S., Li, S., Zhou, J. & Yang, H. Y. Interfacial Molecule Engineering for Reversible Zn Electrochemistry. *ACS Energy Lett.* **8**, 3258–3268 (2023).

42. Wan, J., Wang, R., Liu, Z., Zhang, L., Liang, F., Zhou, T., Zhang, S., Zhang, L., Lu, Q., Zhang, C. & Guo, Z. A Double-Functional Additive Containing Nucleophilic Groups for High-Performance Zn-Ion Batteries. *ACS Nano* **17**, 1610–1621 (2023).

43. Lin, Y., Mai, Z., Liang, H., Li, Y., Yang, G. & Wang, C. Dendrite-free Zn anode enabled by anionic surfactant-induced horizontal growth for highly-stable aqueous Zn-ion pouch cells. *Energy Environ. Sci.* **16**, 687–697 (2023).

44. Cao, L., Li, D., Hu, E., Xu, J., Deng, T., Ma, L., Wang, Y., Yang, X. Q. & Wang, C. Solvation Structure Design for Aqueous Zn Metal Batteries. *J. Am. Chem. Soc.* **142**, 21404–21409 (2020).

45. Huang, C., Zhao, X., Hao, Y., Yang, Y., Qian, Y., Chang, G., Zhang, Y., Tang, Q., Hu, A. & Chen, X. Self-Healing SeO_2_ Additives Enable Zinc Metal Reversibility in Aqueous ZnSO_4_ Electrolytes. *Adv. Funct. Mater.* **32**, 2112091 (2022).

46. Yao, R., Zhao, Y., Wang, L., Xiao, C., Kang, F., Zhi, C. & Yang, C. A corrosion-free zinc metal battery with an ultra-thin zinc anode and high depth of discharge. *Energy Environ. Sci.* **17**, 3112–3122 (2024).
